# Supplementary material for: Preparation of Sesquiterpene Lactone Derivatives: Cytotoxic Activity and Selectivity of Action
Source: Molecules. 2019 Mar 20;24(6):1113. doi: 10.3390/molecules24061113 (PMC6471591; doi:10.3390/molecules24061113)
Supplement: Supplementary file 1 [file molecules-24-01113-s001.pdf]

Supporting Information

Oxygenated and oxo-nitrogenated derivatives of sesquiterpene lactones: cytotoxic activity and selectivity of action

María F. Beer[1, 2], Augusto E. Bivona[3], Andrés Sánchez Alberti[3], Natacha Cerny[4], Guillermo F. Reta[1], Víctor S. Martín[5], José M. Padrón[3], Emilio Malchiodi[3], Valeria Sülsen[2,6,ψ], and Osvaldo J. Donadel[1,*,ψ]

[1] INTEQUI-CONICET, Facultad de Química, Bioquímica y Farmacia, Universidad Nacional de San Luis, Almirante Brown 1445 - CP D5700HGC - San Luis, Argentina.

[2] CONICET – Universidad de Buenos Aires. Instituto de Química y Metabolismo del Fármaco - CONICET (IQUIMEFA), Junín 956 2°P (1113). Buenos Aires, Argentina.

[3] Universidad de Buenos Aires, Facultad de Farmacia y Bioquímica, Cátedra de Inmunología. Junín 956 2°P (1113). Buenos Aires, Argentina. Instituto de Estudios de la Inmunidad Humoral (IDEHU), UBA-CONICET. Junín 956 4°P (1113), Buenos Aires, Argentina; CONICET- Universidad de Buenos Aires. Instituto de Microbiología y Parasitología Médica - CONICET (IMPaM), Facultad de Medicina. Paraguay 2155. Piso 13, Buenos Aires, Argentina.

[4] CONICET - Universidad Nacional de Luján. Instituto de Ecología y Desarrollo Sustentable (INEDES). Ruta 5 y Avenida Constitución - (6700). Luján, Argentina.

[5] Instituto Universitario de Bio-Orgánica Antonio González (IUBO-AG), Universidad de La Laguna, Avda. Astrofísico Francisco Sánchez 2, 38206 La Laguna, Spain.

[6] Universidad de Buenos Aires, Cátedra de Farmacognosia, Facultad de Farmacia y Bioquímica, Junín 956 2°P (1113). Buenos Aires, Argentina

* Correspondence: odonadel@gmail.com; Tel.: +54-266-452-0300, Ext 3151; vsulsen@ffyb.uba.ar; Tel.: +54-11-5287-4272.

Table of contents

1.	NMR spectra of natural sesquiterpene lactones cumanin (**1**), helenalin (**2**) and hymenin (**3**)	Pages S2−S4
2.	NMR spectra of cumanin derivatives (**4**-**11**)	Pages S5−S15
3.	NMR spectra of helenalin derivatives (**12**-**14**)	Pages S16−S18
4.	NMR spectra of hymenin derivative (**15**)	Pages S19
5.	HRMS-ES of compounds **1**-**15**	Pages S20−S27

1. NMR spectra of natural sesquiterpene lactones cumanin (1), helenalin (2) and hymenin (3)

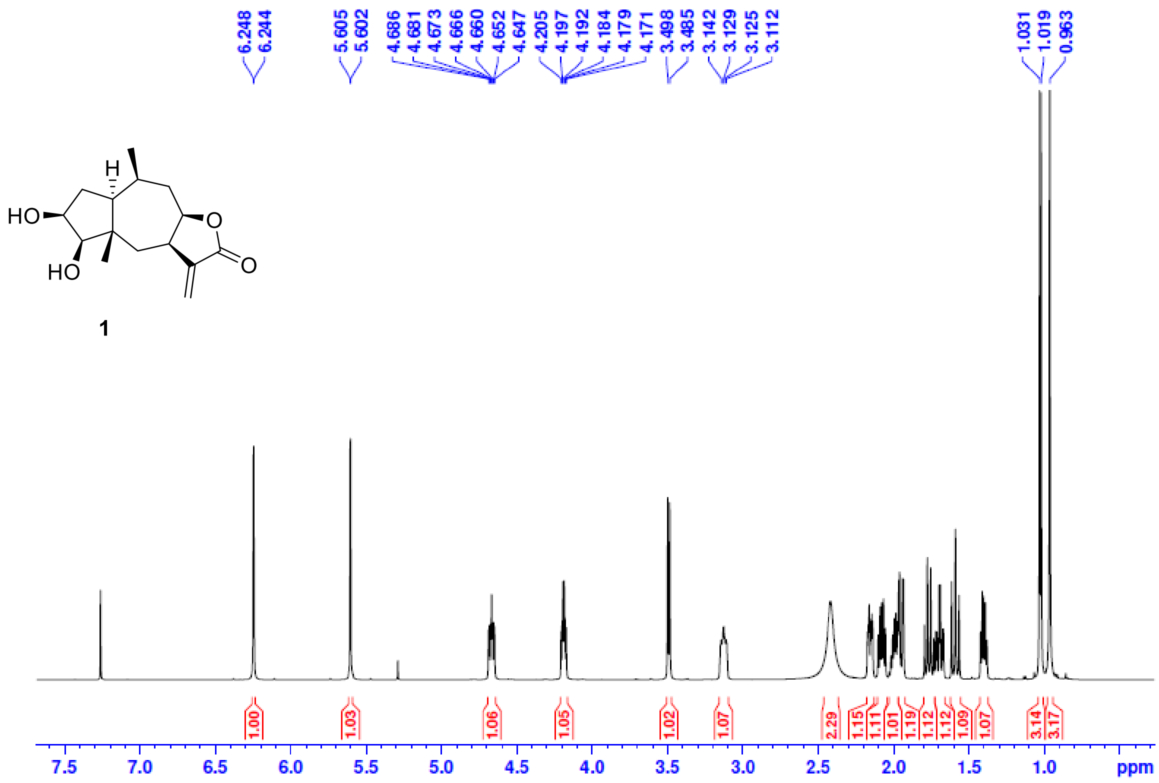

Figure S1. ^{1}H-NMR of **1**

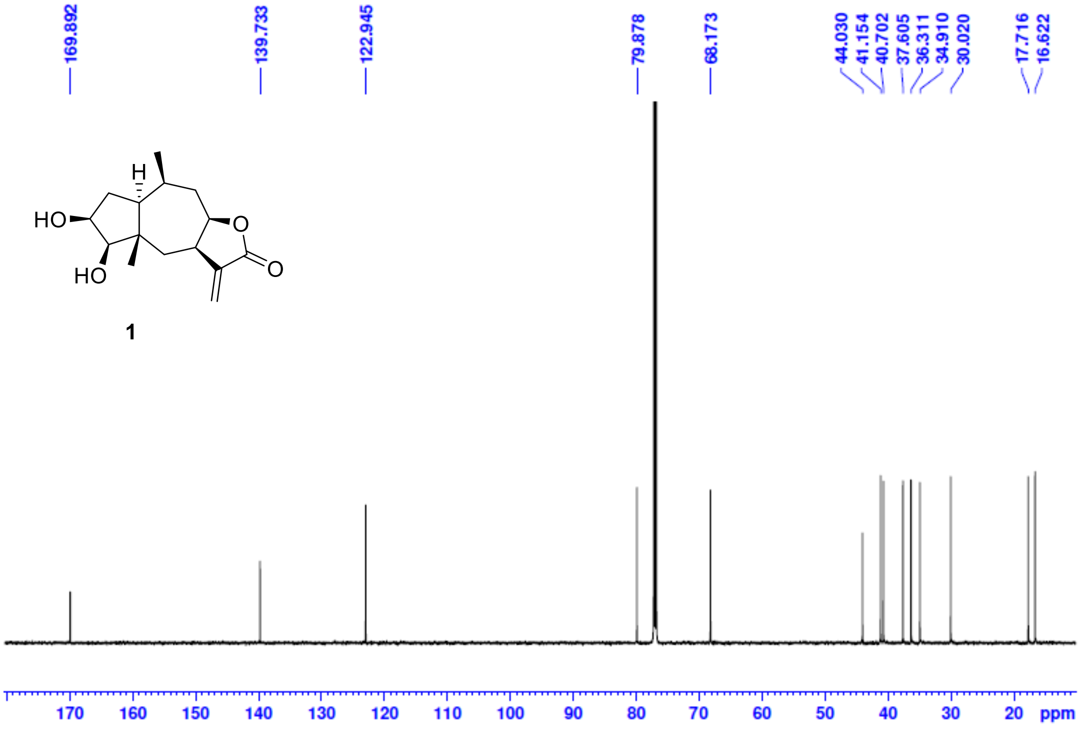

Figure S2. ^{13}C-NMR of **1**

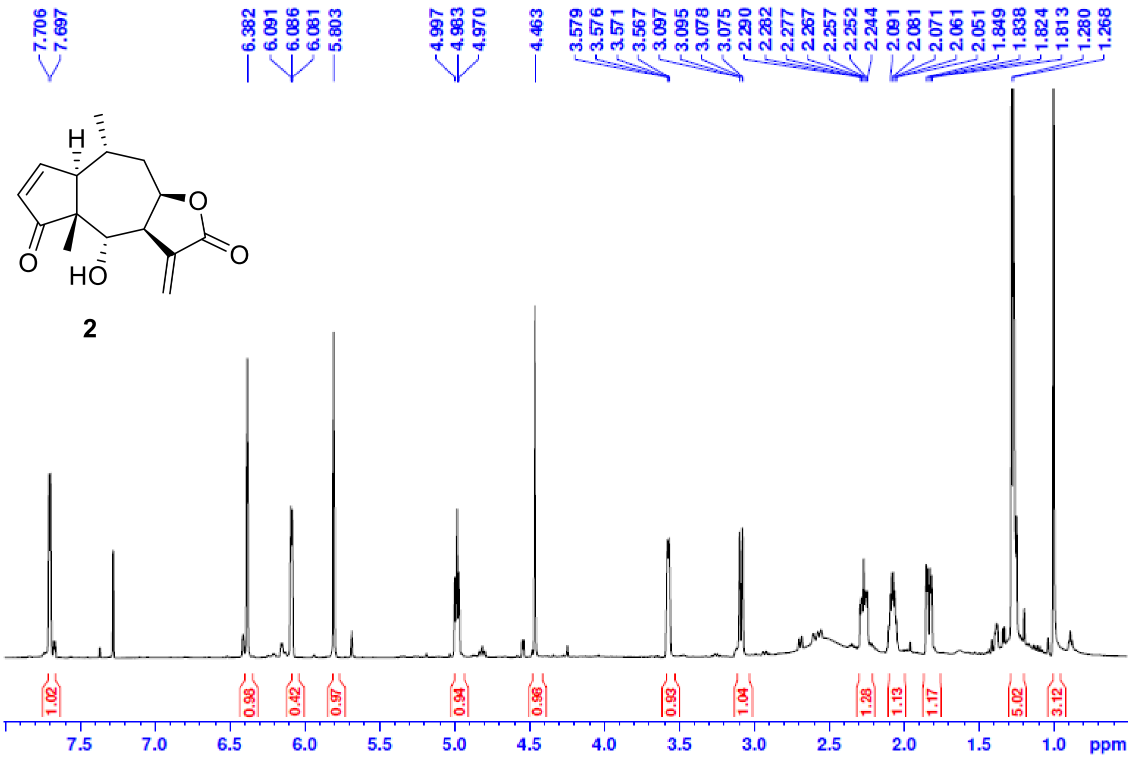

Figure S3. ^{1}H-NMR of **2**

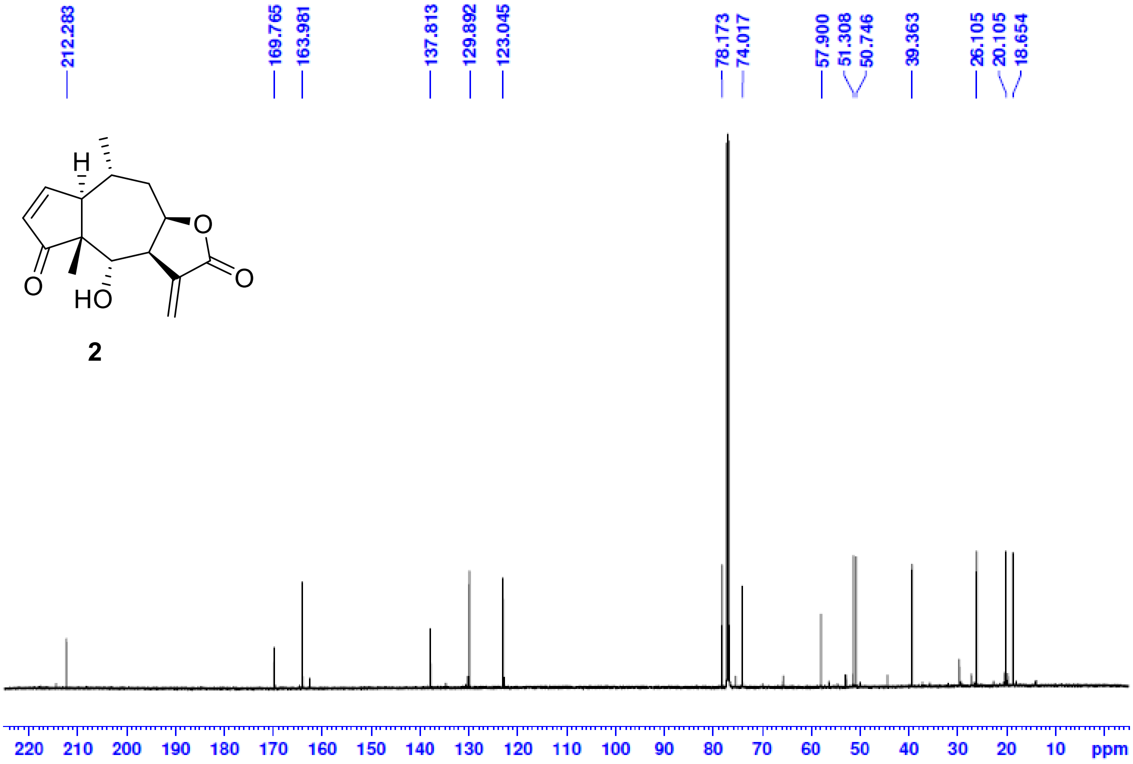

Figure S4. ^{13}C-NMR of **2**

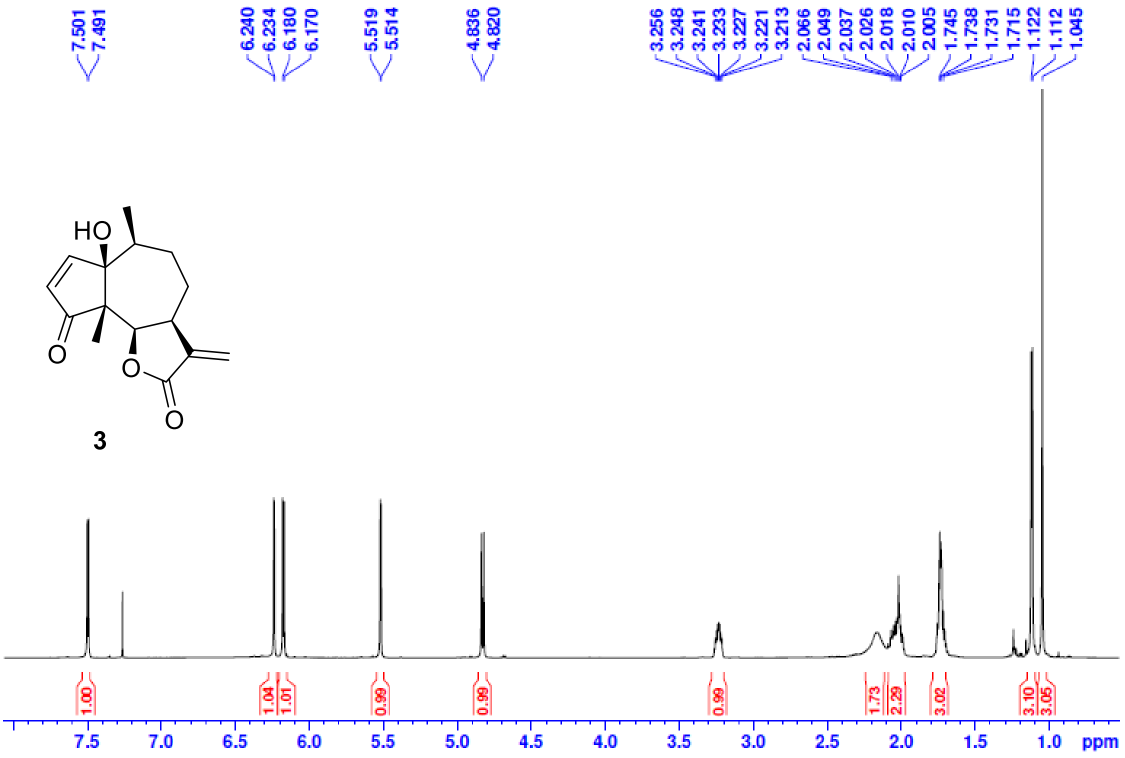

Figure S5. ^{1}H-NMR of **3**

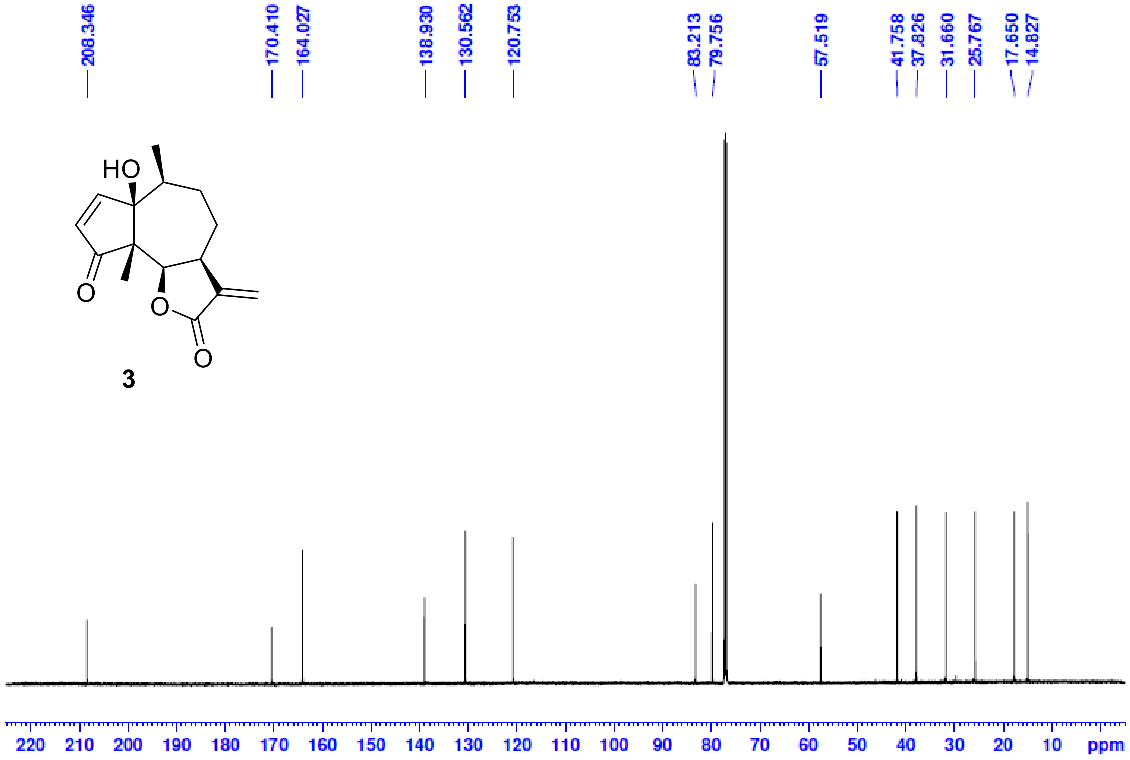

Figure S6. ^{13}C-NMR of **3**

2. NMR spectra of cumanin derivatives

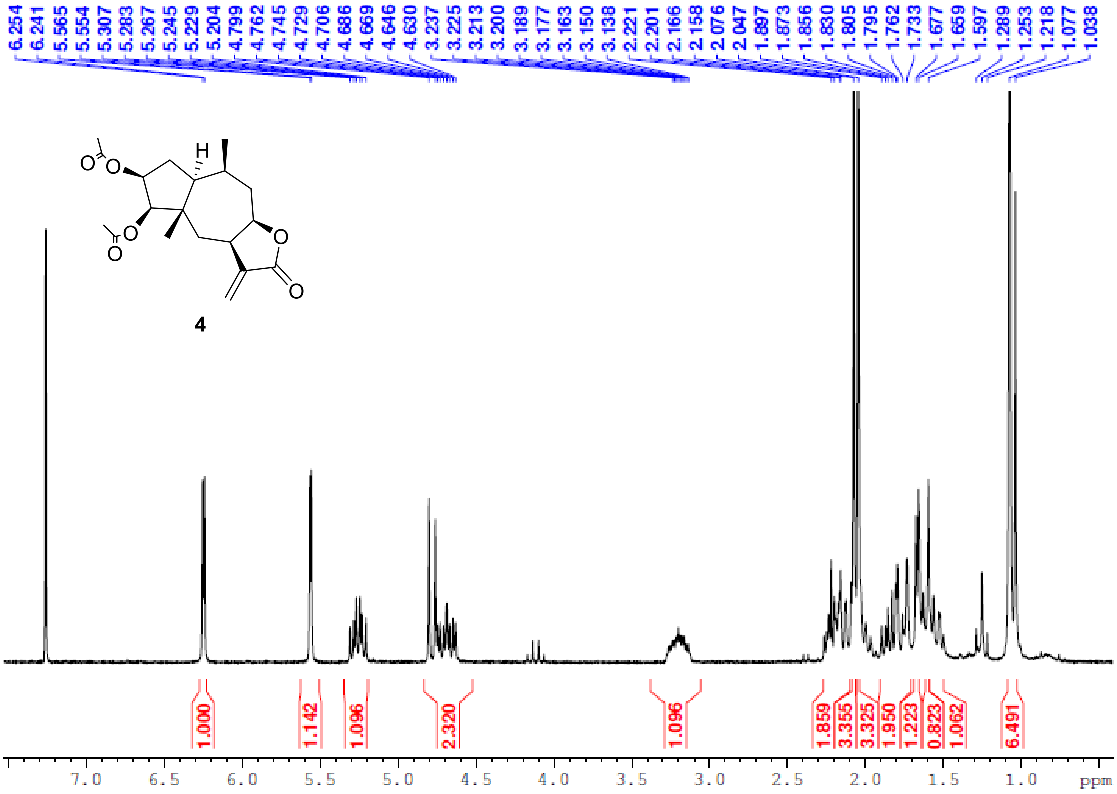

Figure S7. ^{1}H-NMR of **4**

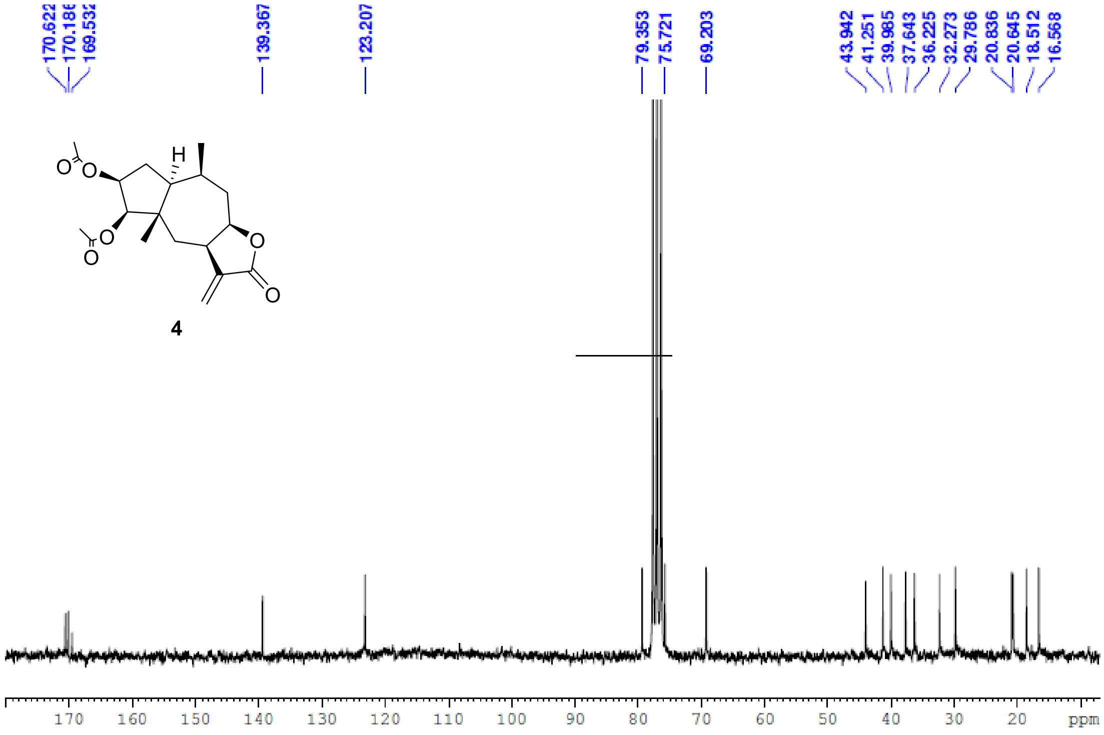

Figure S8. ^{13}C-NMR of **4**

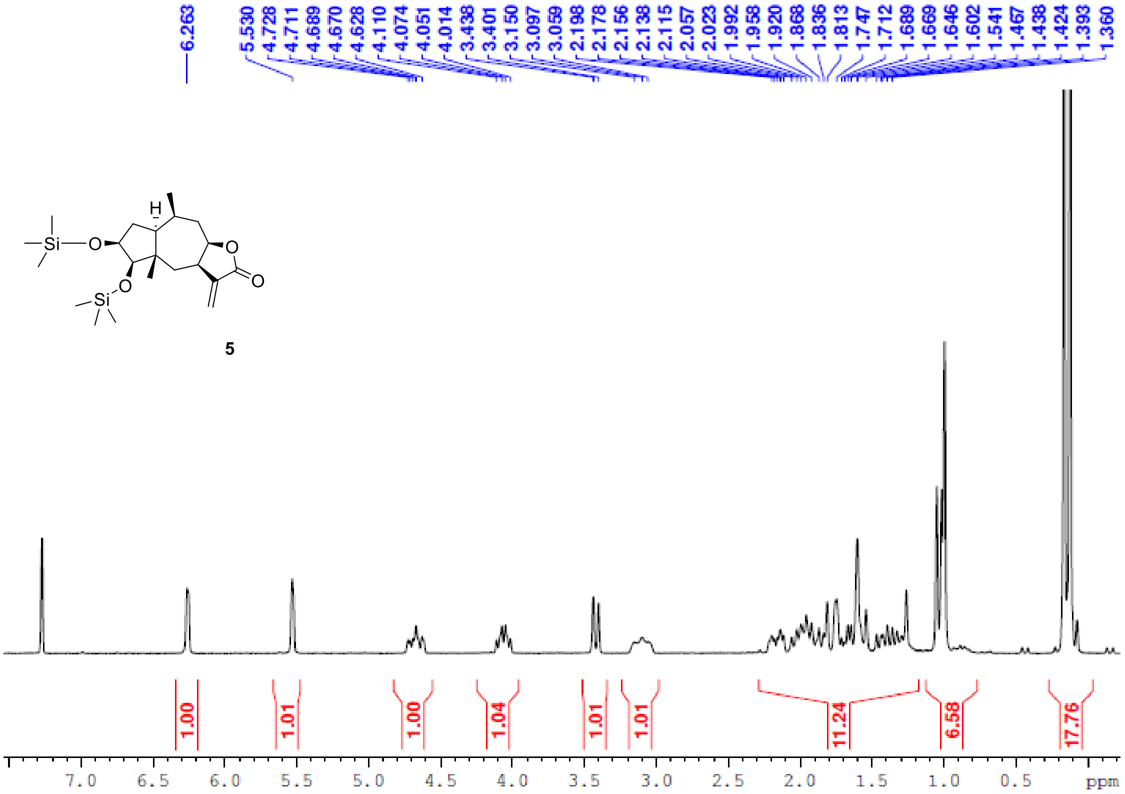

Figure S9. ^{1}H-NMR of **5**

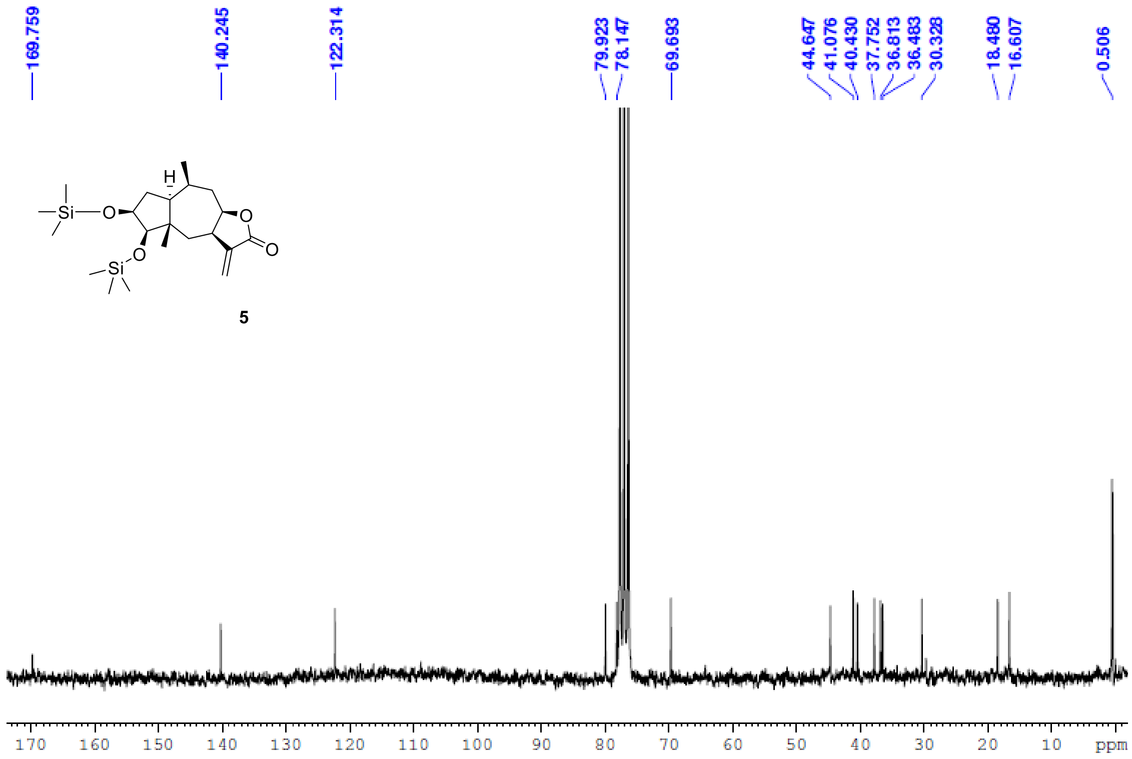

Figure S10. ^{13}C-NMR of **5**

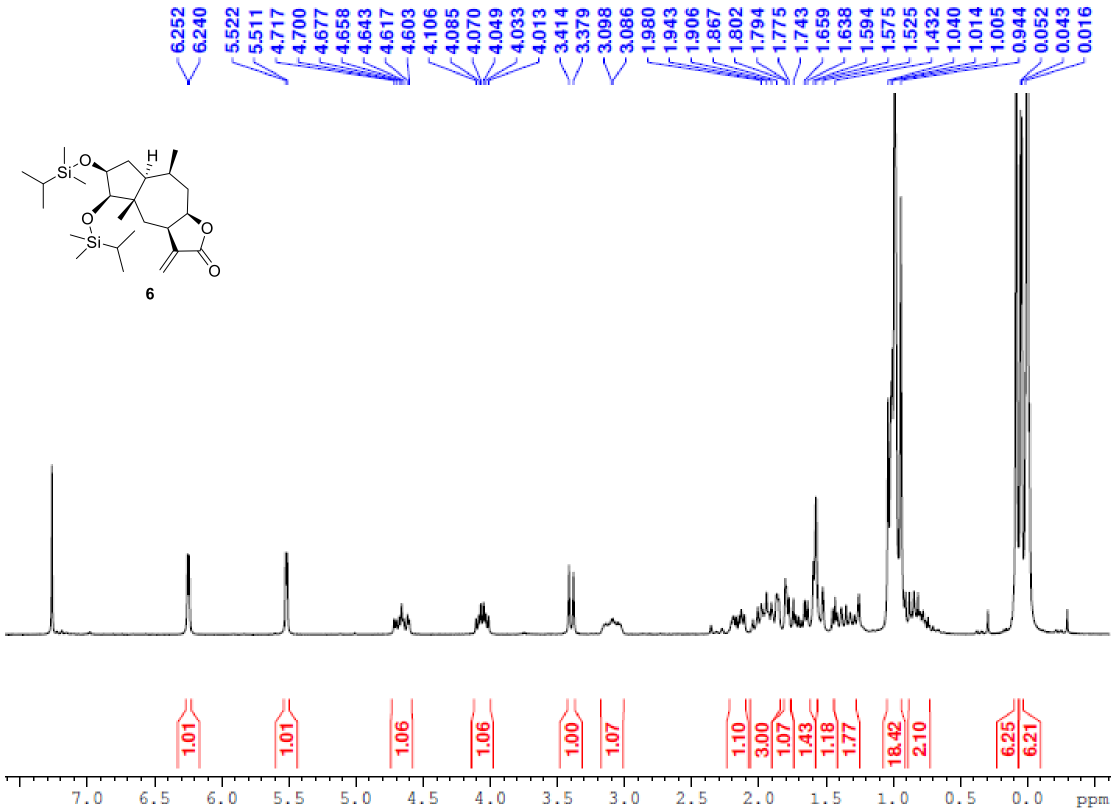

Figure S11. ^{1}H-NMR of **6**

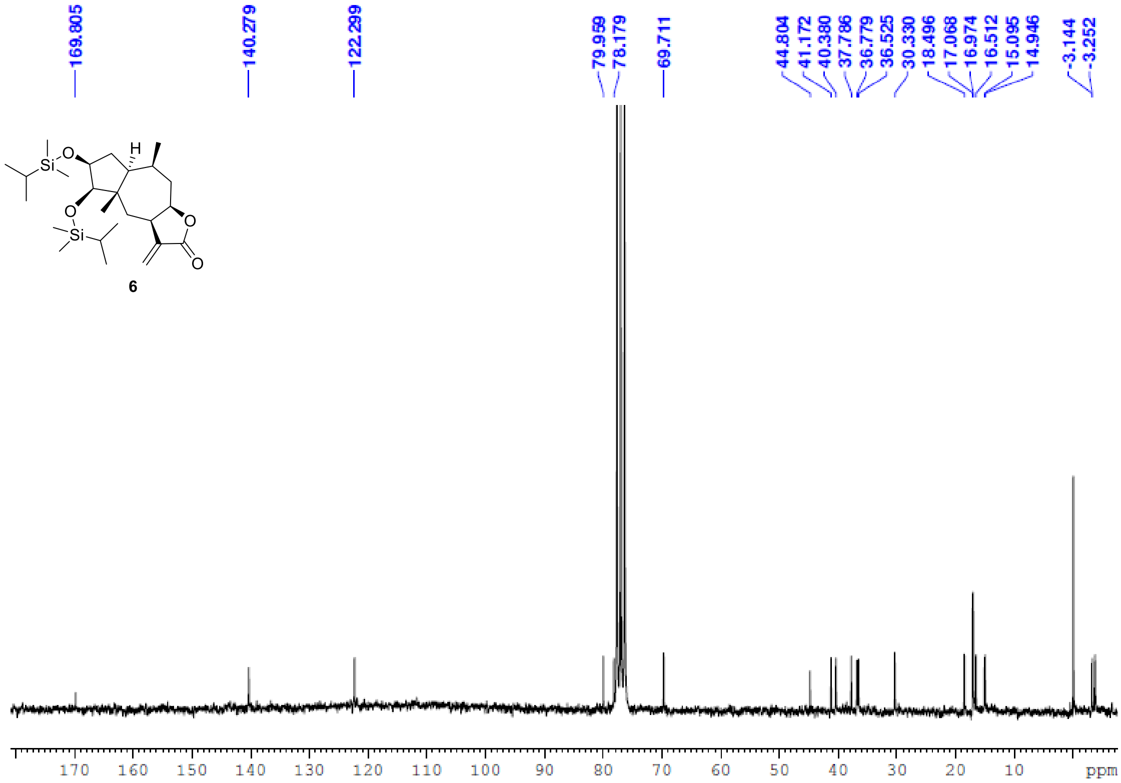

Figure S12. ^{13}C-NMR of **6**

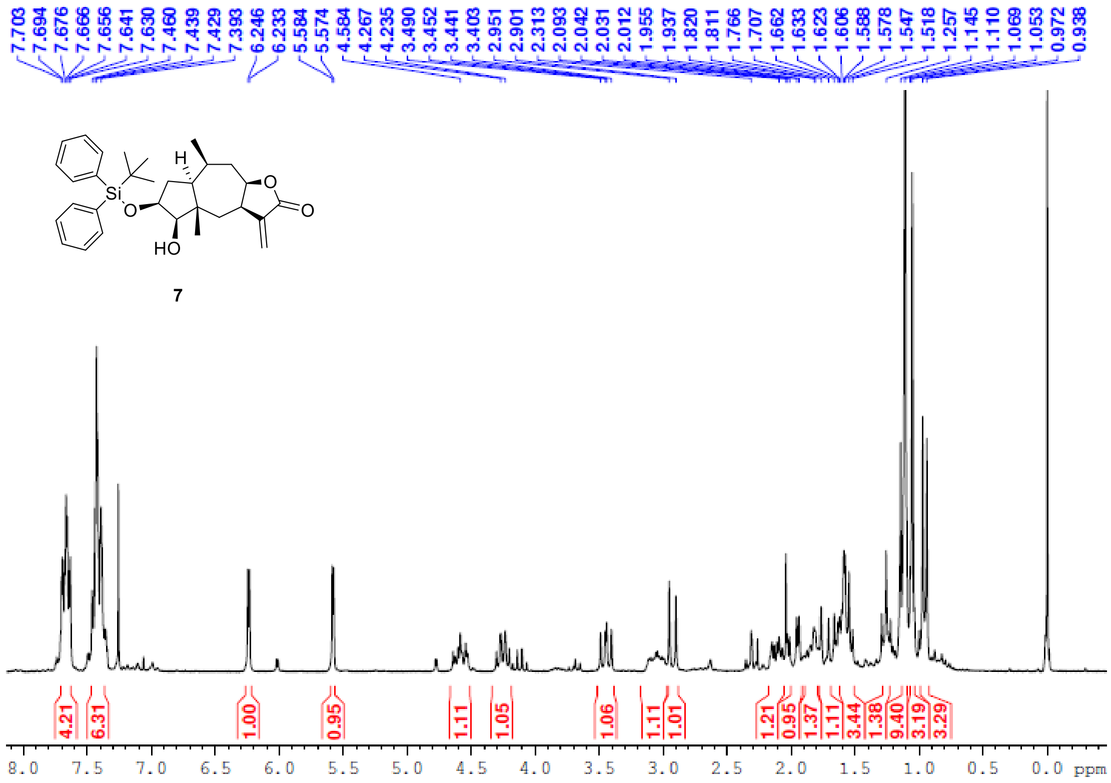

Figure S13. ^{1}H-NMR of **7**

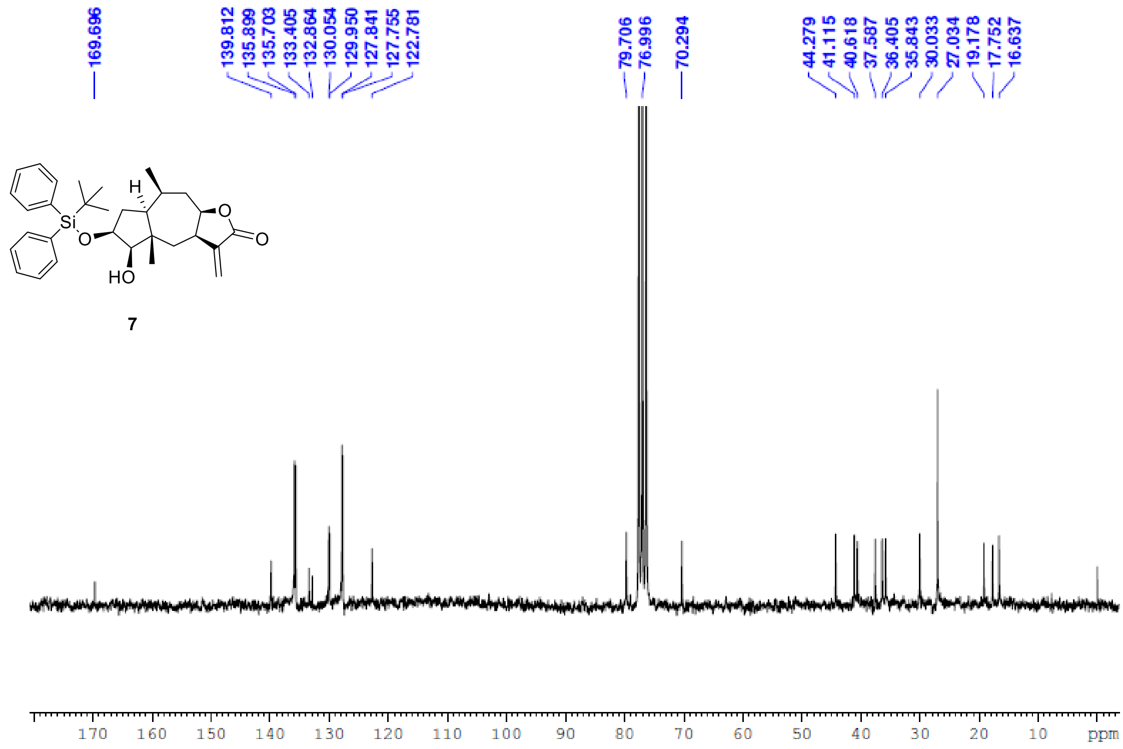

Figure S14. ^{13}C-NMR of **7**

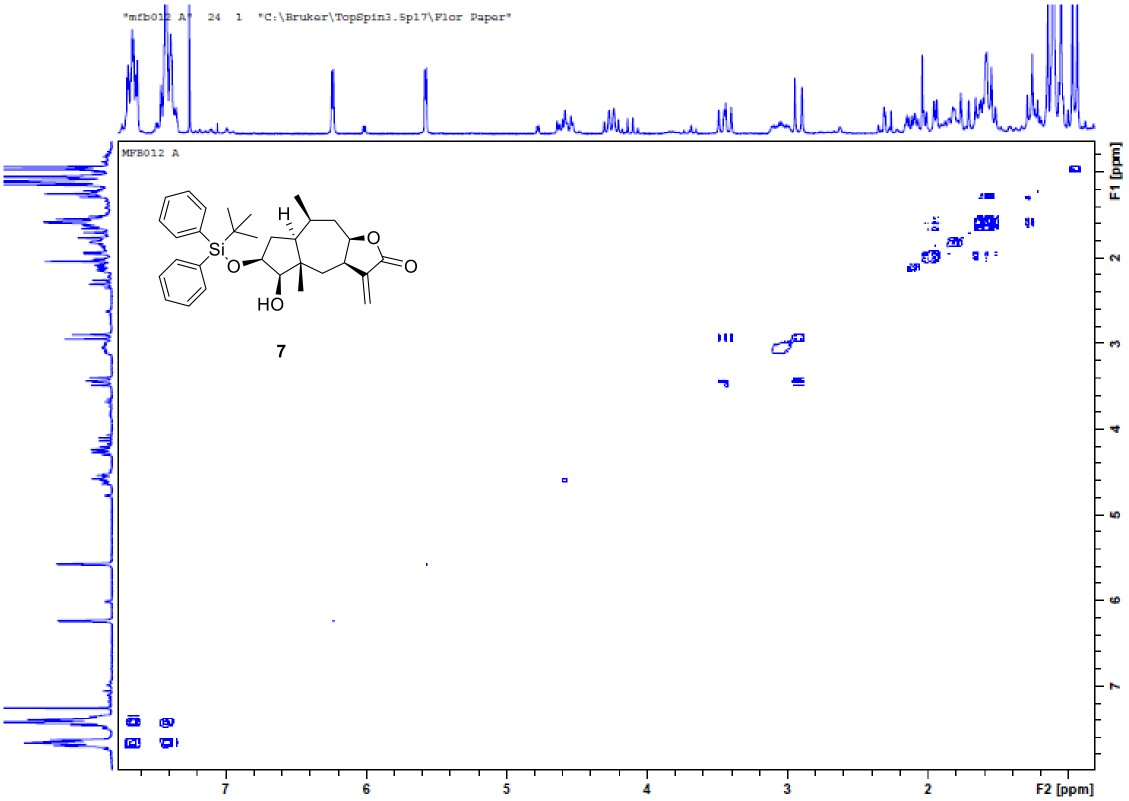

Figure S15. COSY of **7**

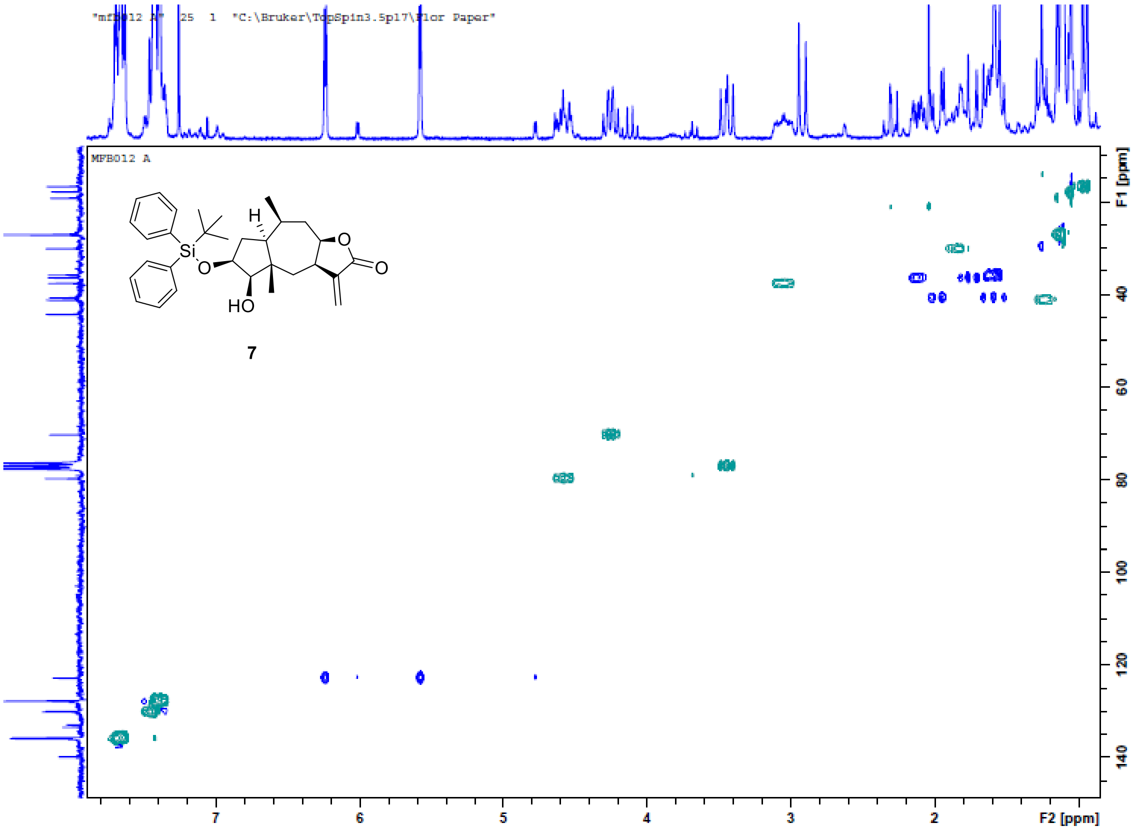

Figure S16. HSQC of **7**

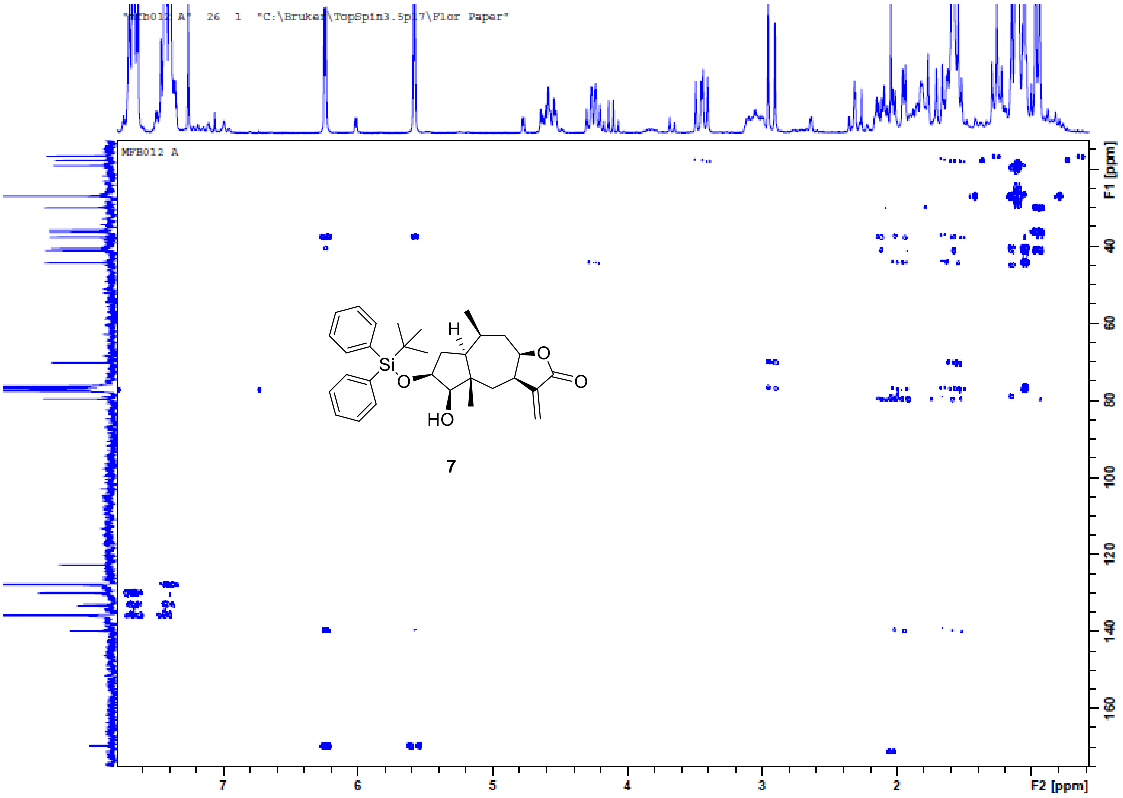

Figure S17. HMBC of **7**

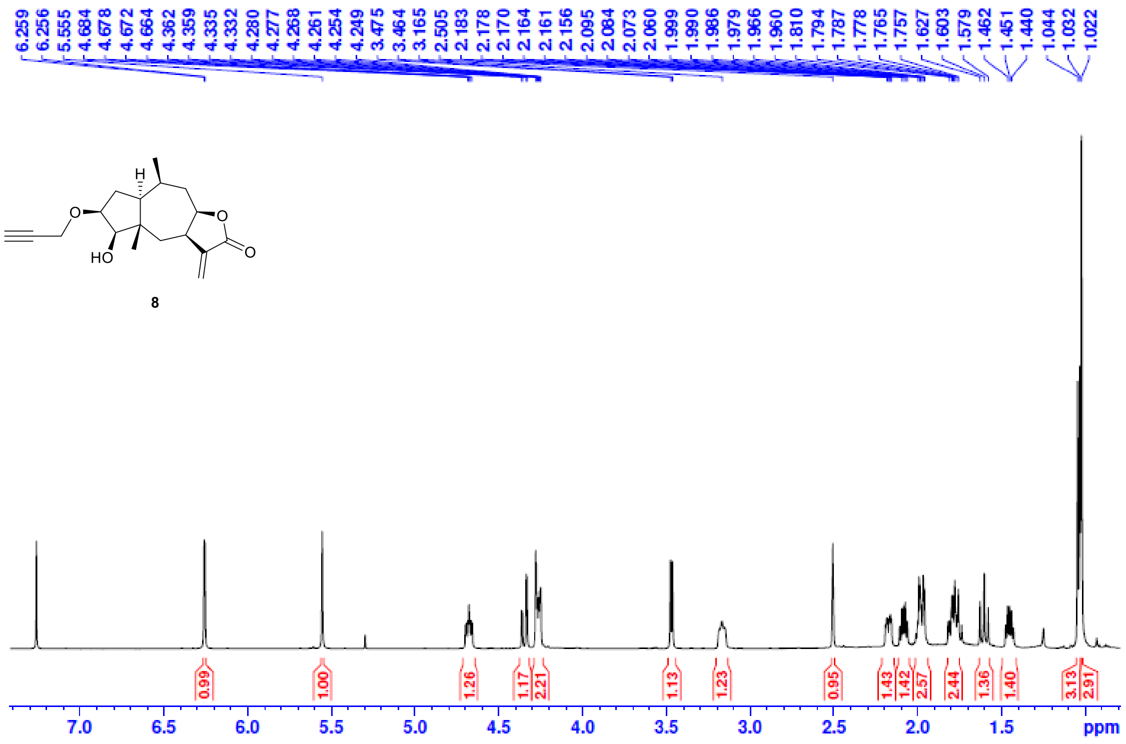

Figure S18. ^{1}H-NMR of **8**

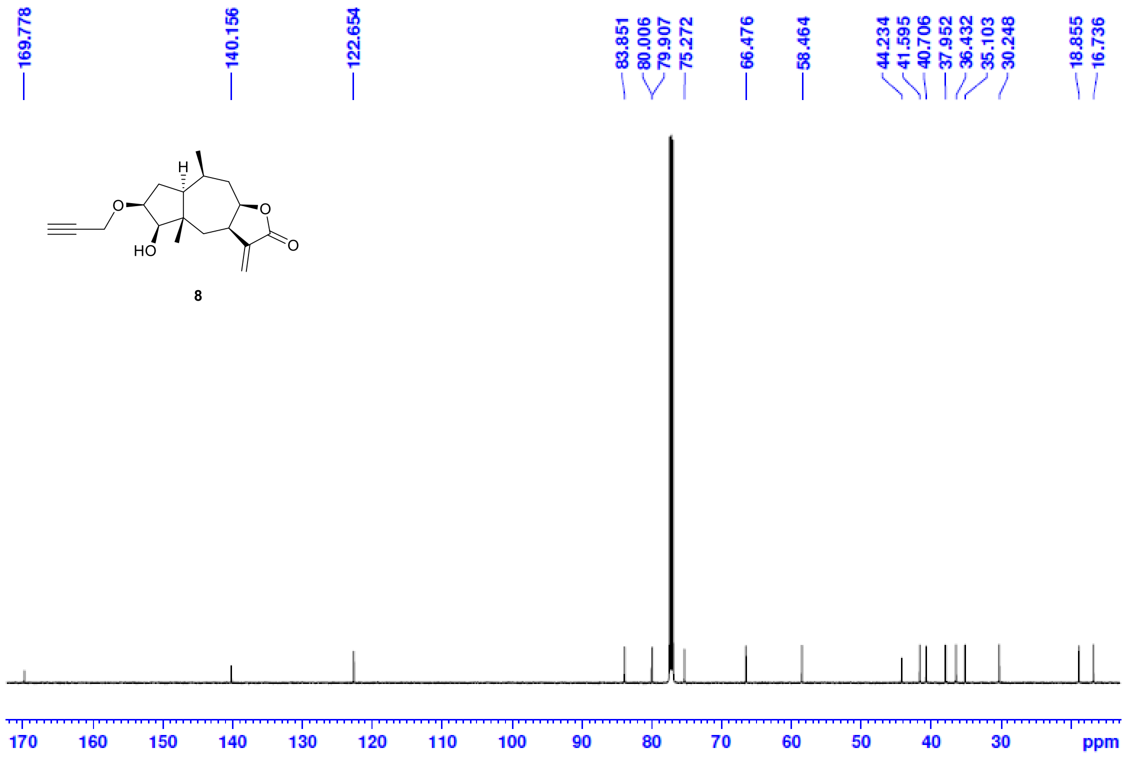

Figure S19. ^{13}C-NMR of **8**

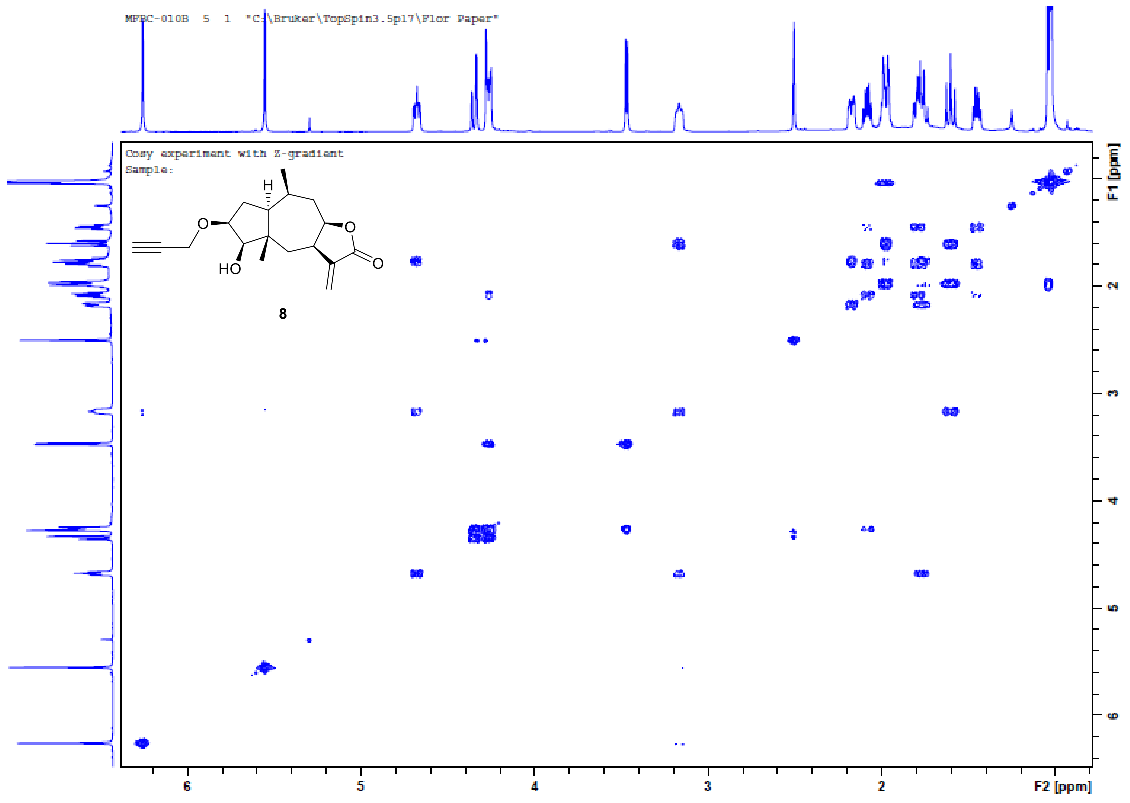

Figure S20. COSY of **8**

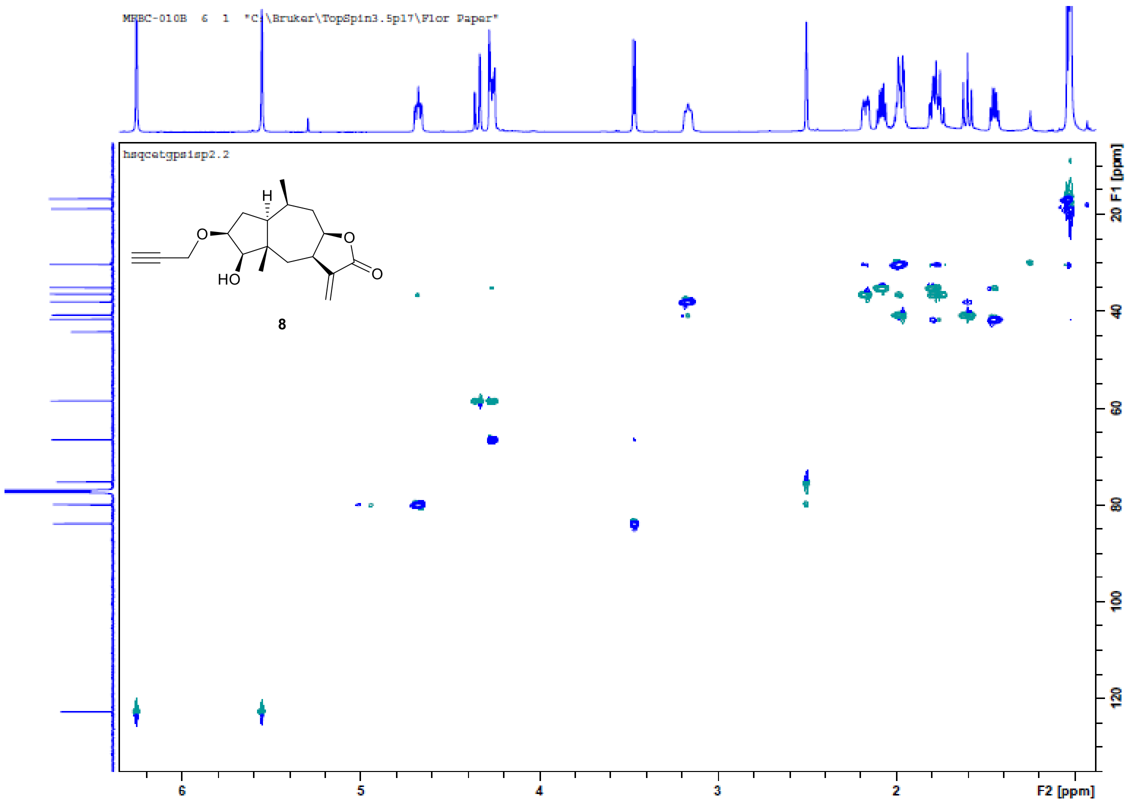

Figure S21. HSQC of **8**

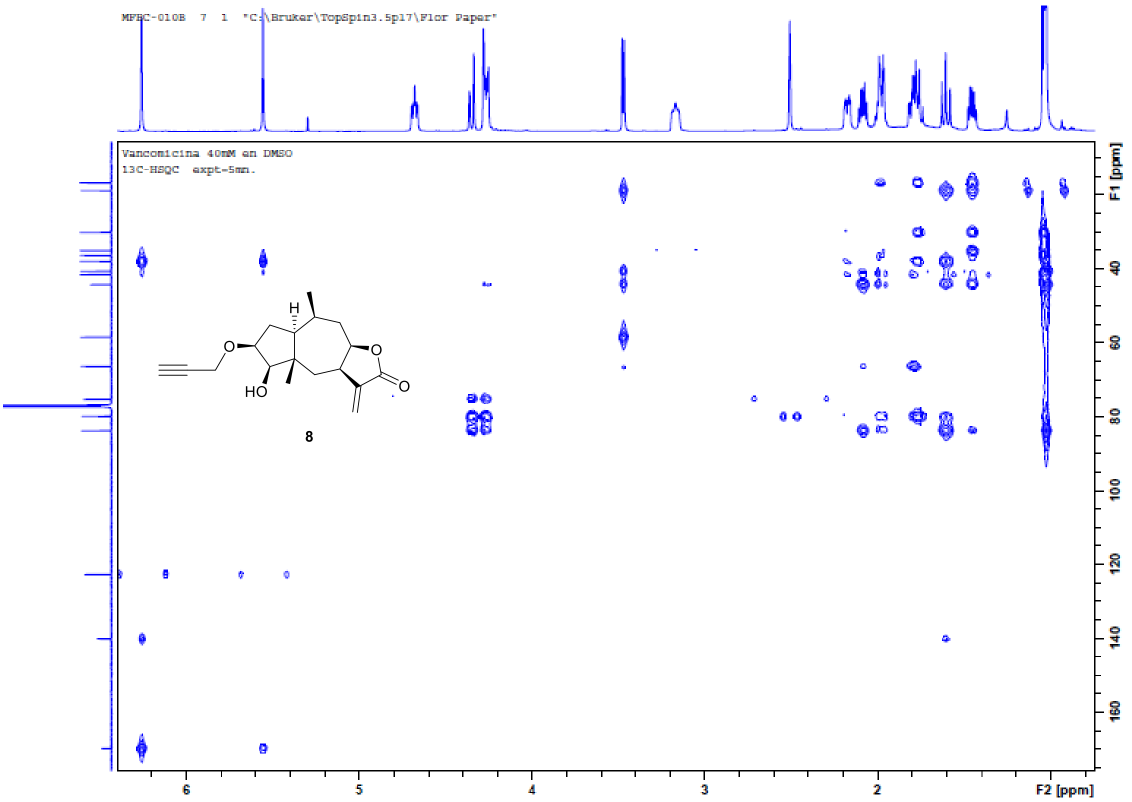

Figure S22. HSQC of **8**

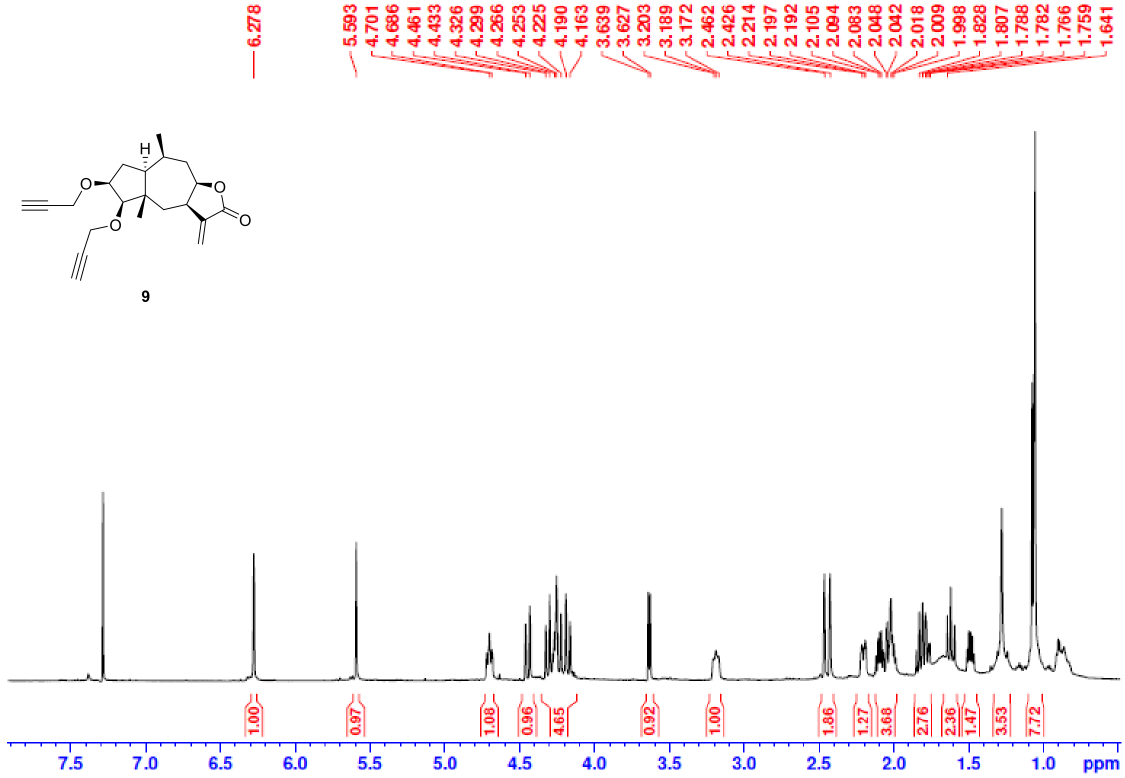

Figure S23. ^{1}H-NMR of **9**

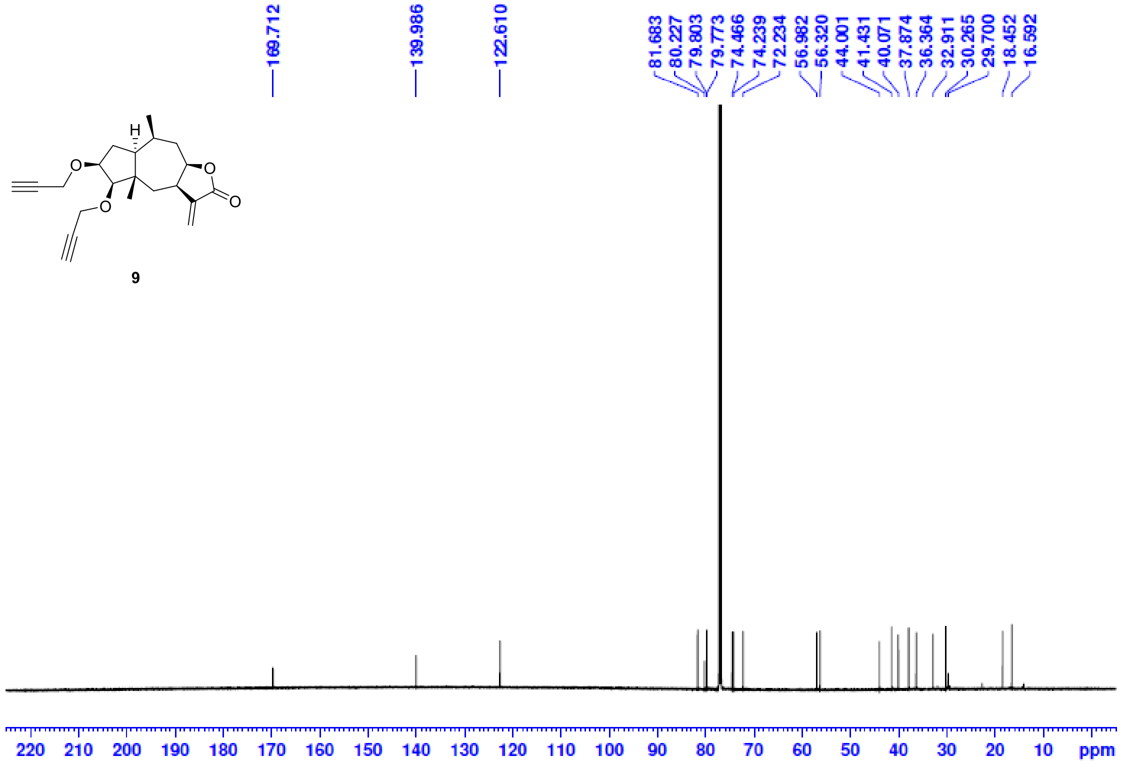

Figure S24. ^{13}C-NMR of **9**

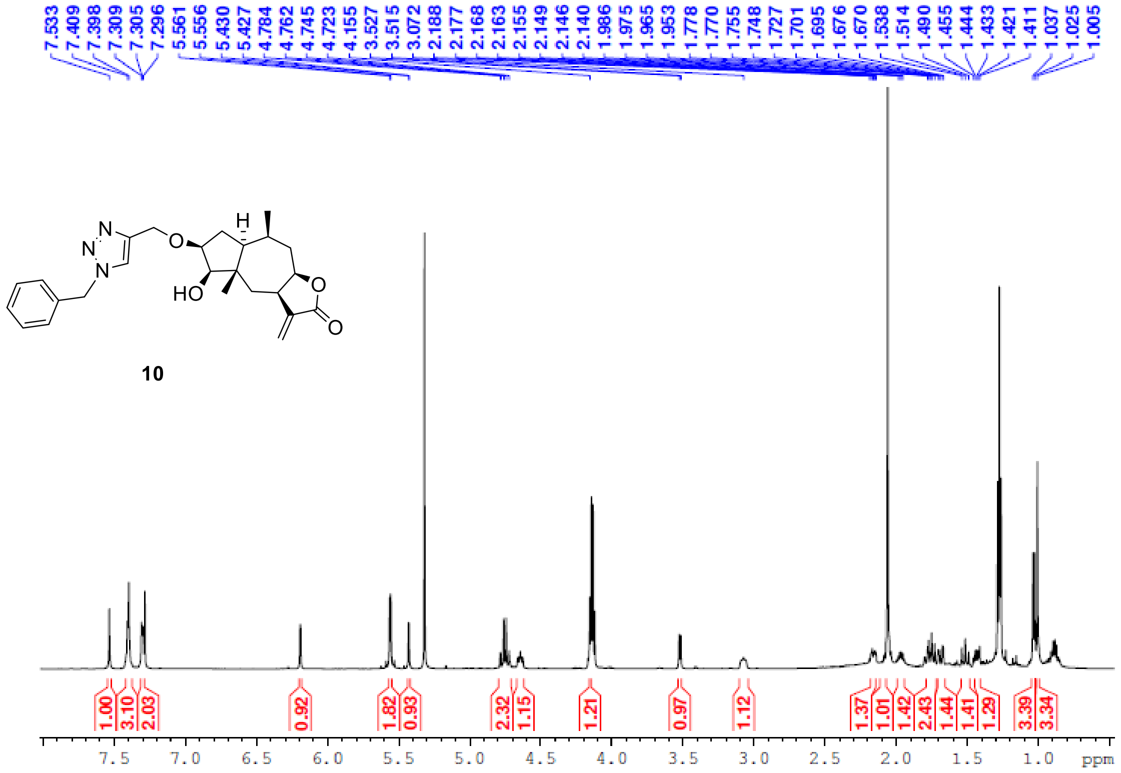

Figure S25. ^{1}H-NMR of **10**

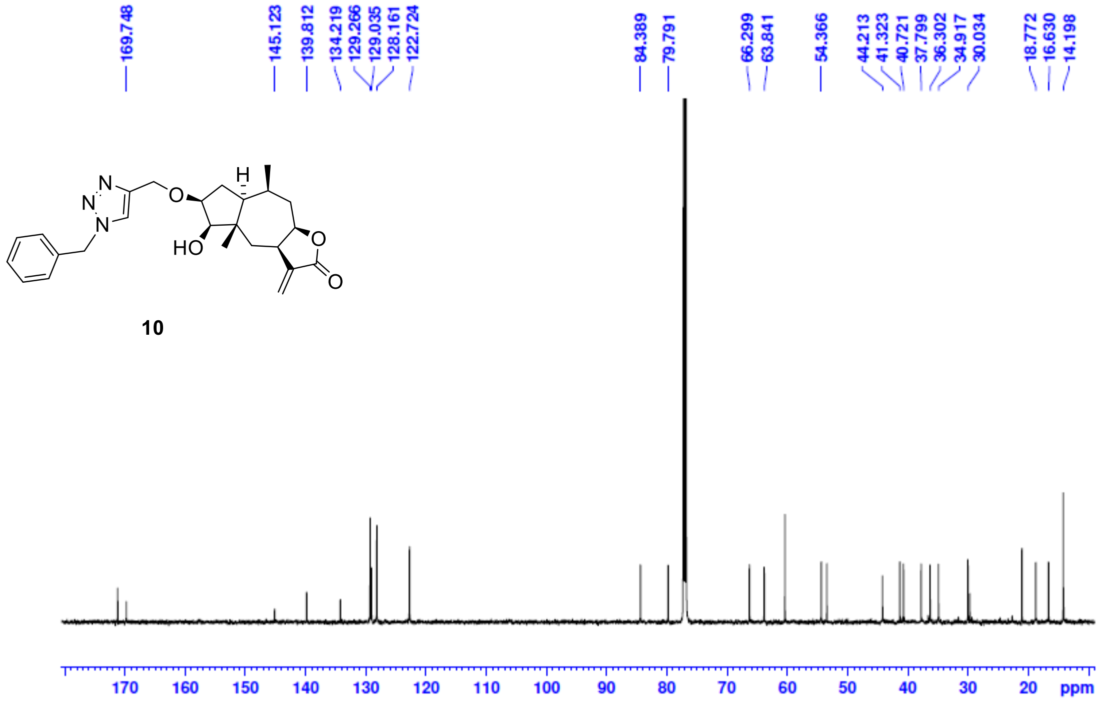

Figure S26. ^{13}C-NMR of **10**

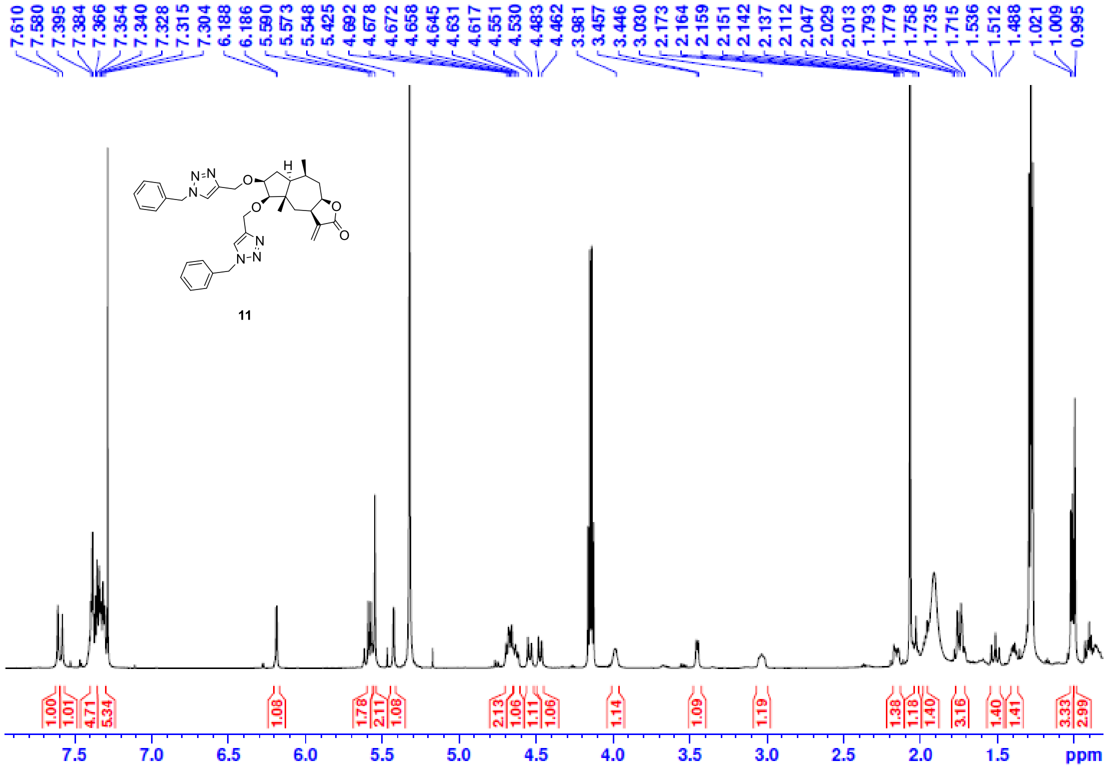

Figure S27. ^{1}H-NMR of **11**

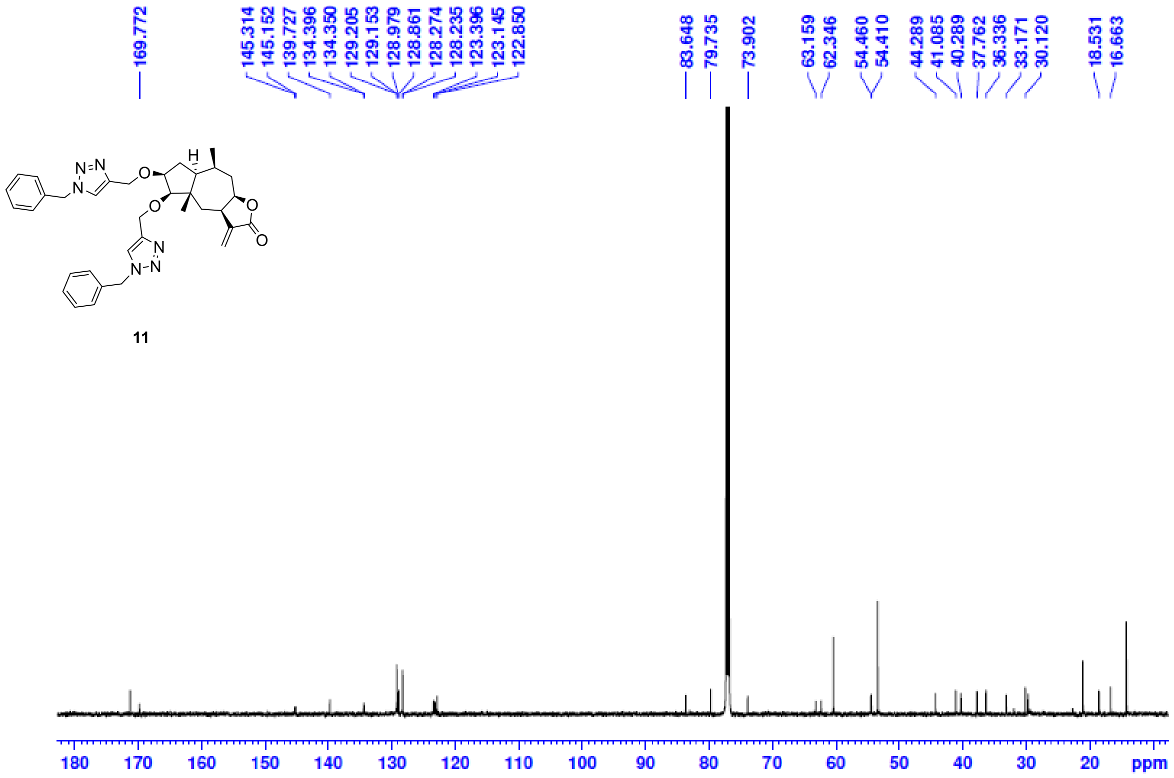

Figure S28. ^{13}C-NMR of **11**

3. NMR spectra of helenalin derivatives

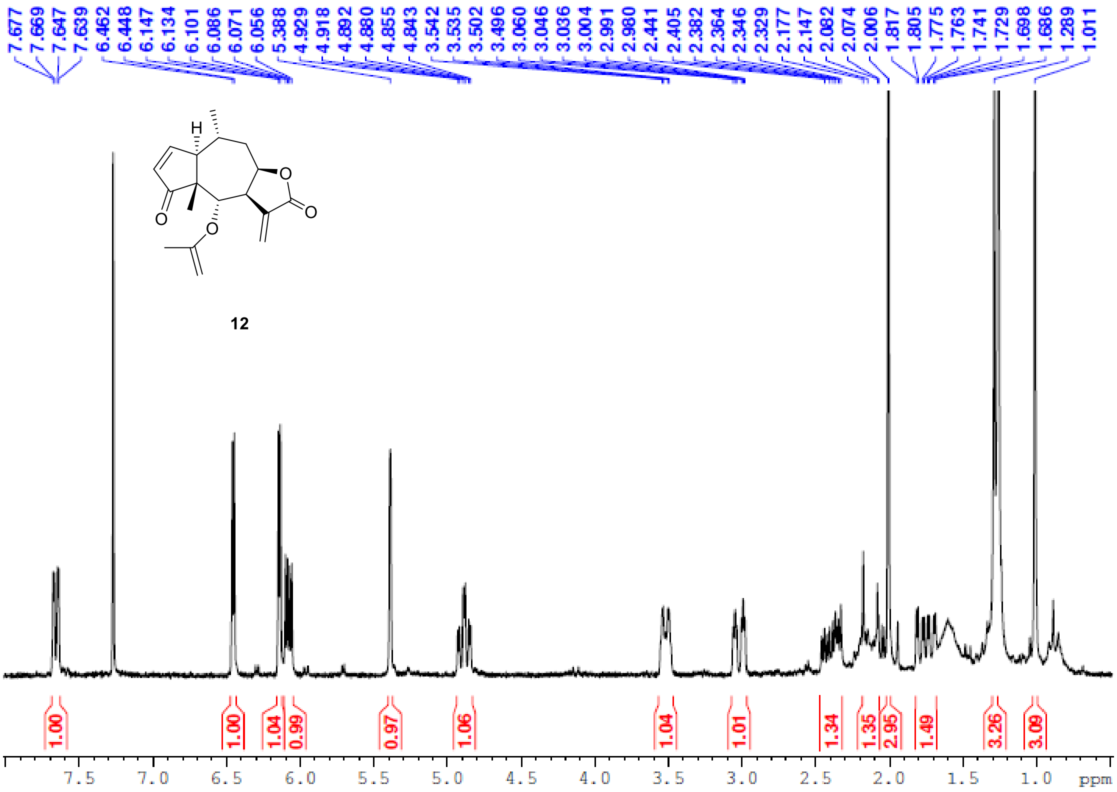

Figure S29. ^{1}H-NMR of **12**

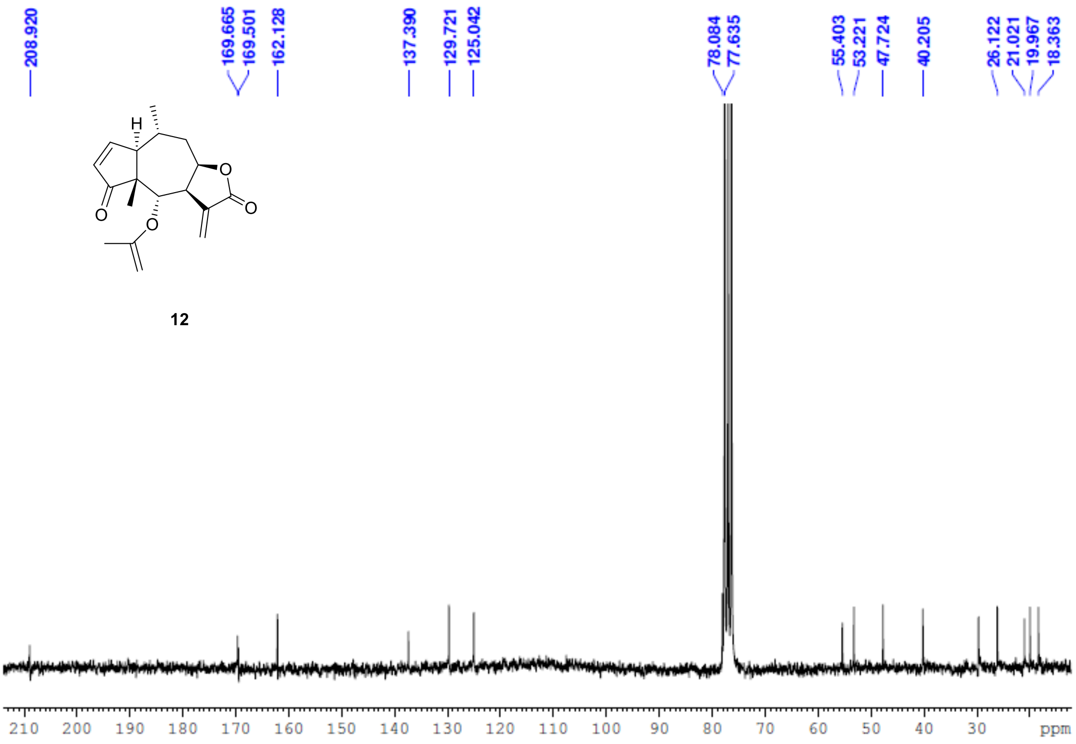

Figure S30. ^{13}C-NMR of **12**

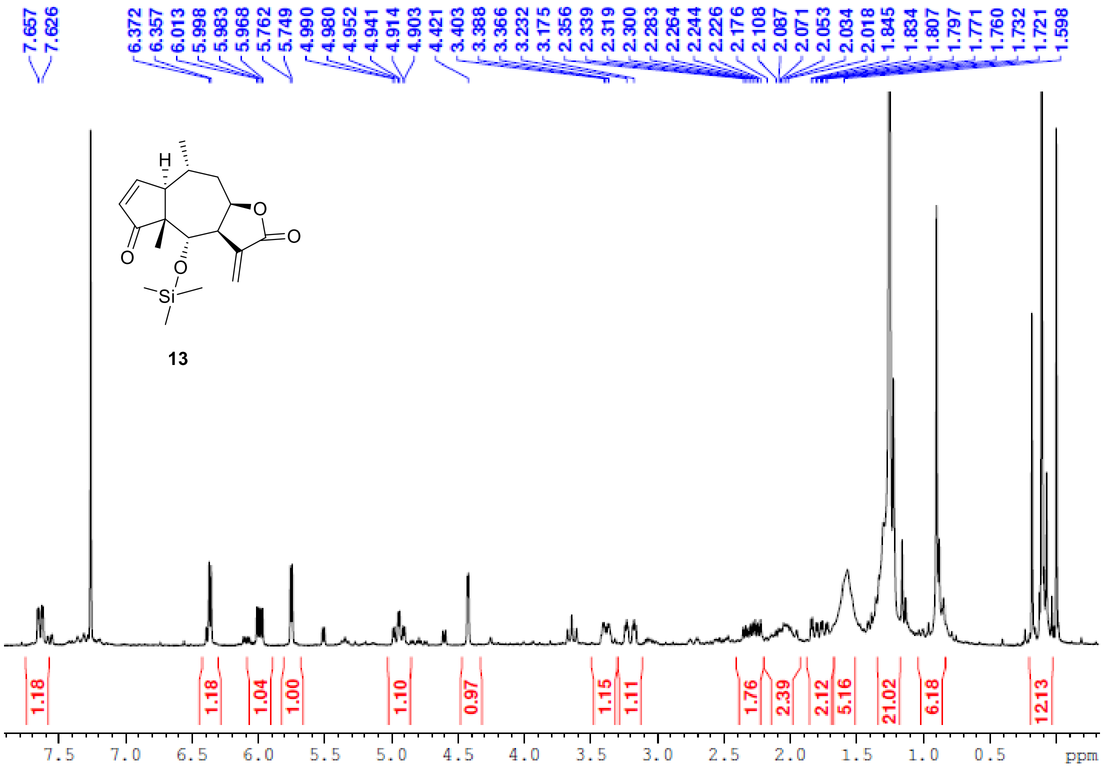

Figure S31. ^{1}H-NMR of **13**

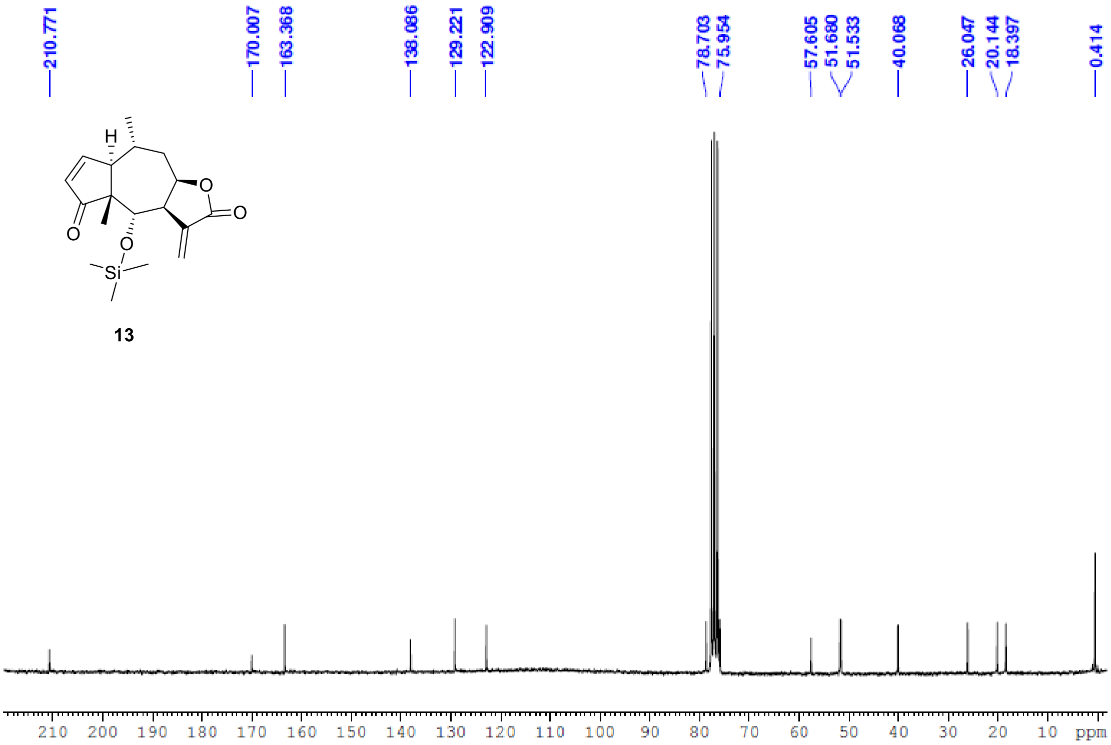

Figure S32. ^{13}C-NMR of **13**

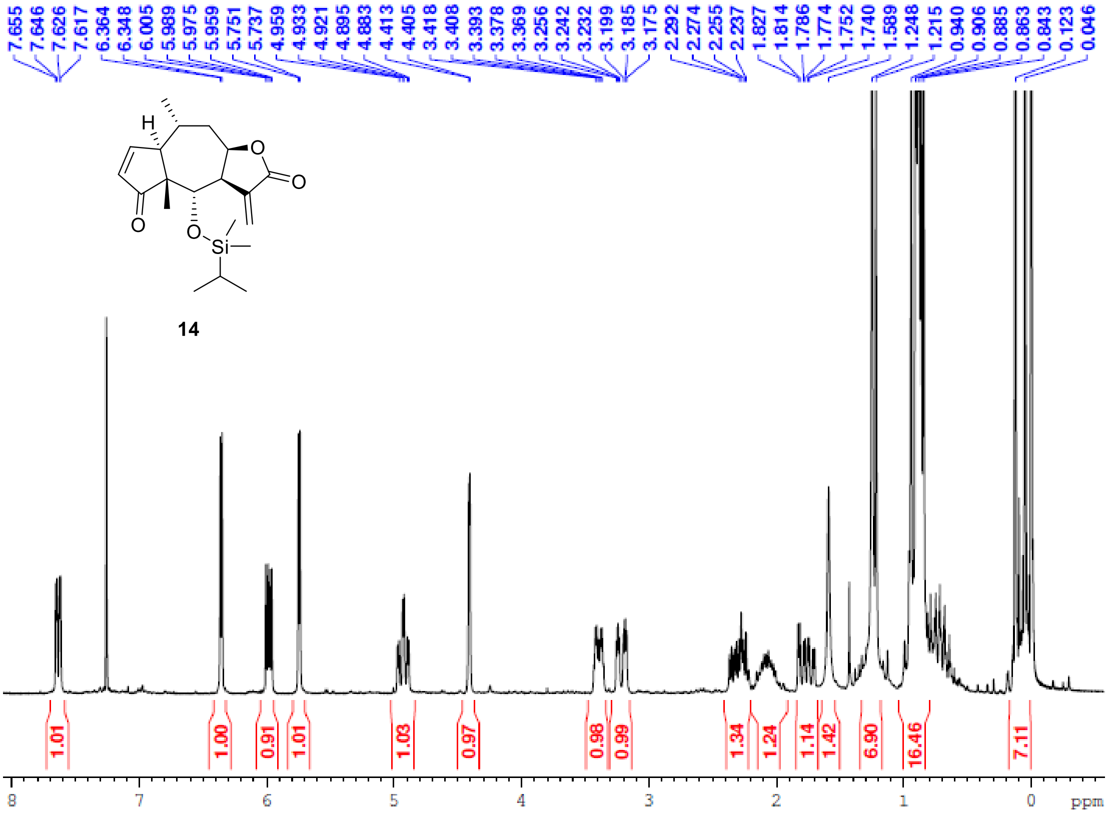

Figure S33. ^{1}H-NMR of **14**

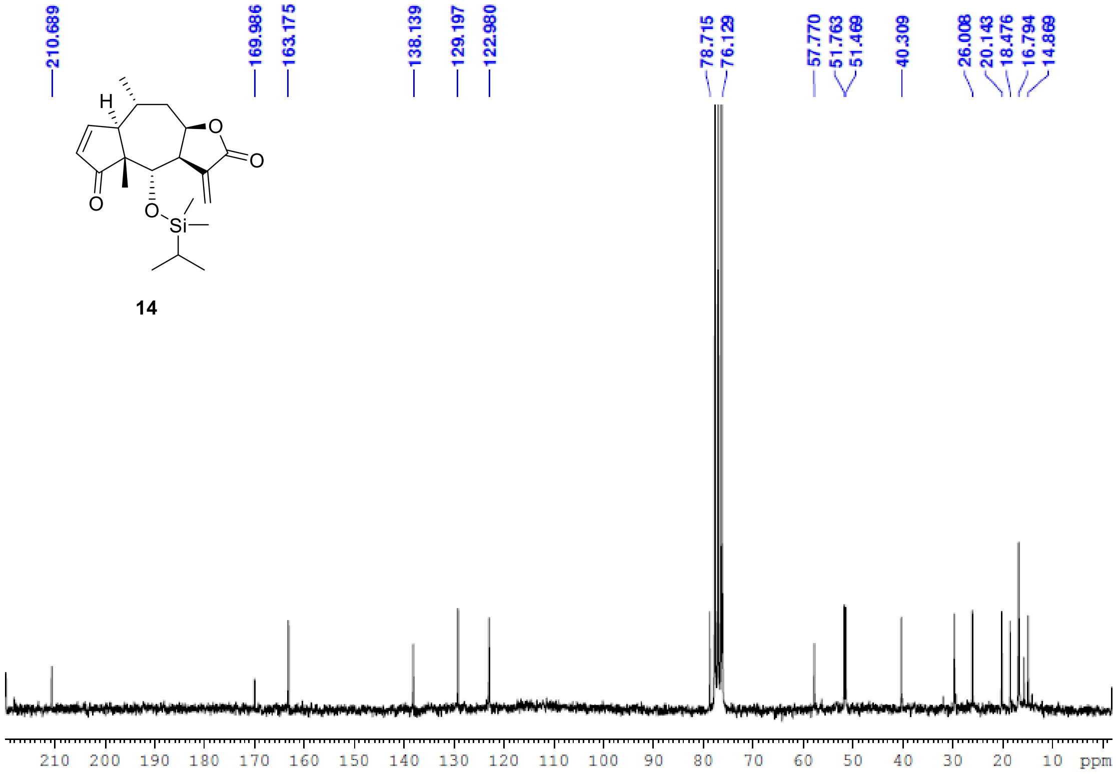

Figure S34. ^{13}C-NMR of **14**

4. NMR spectra of hymenin derivatives

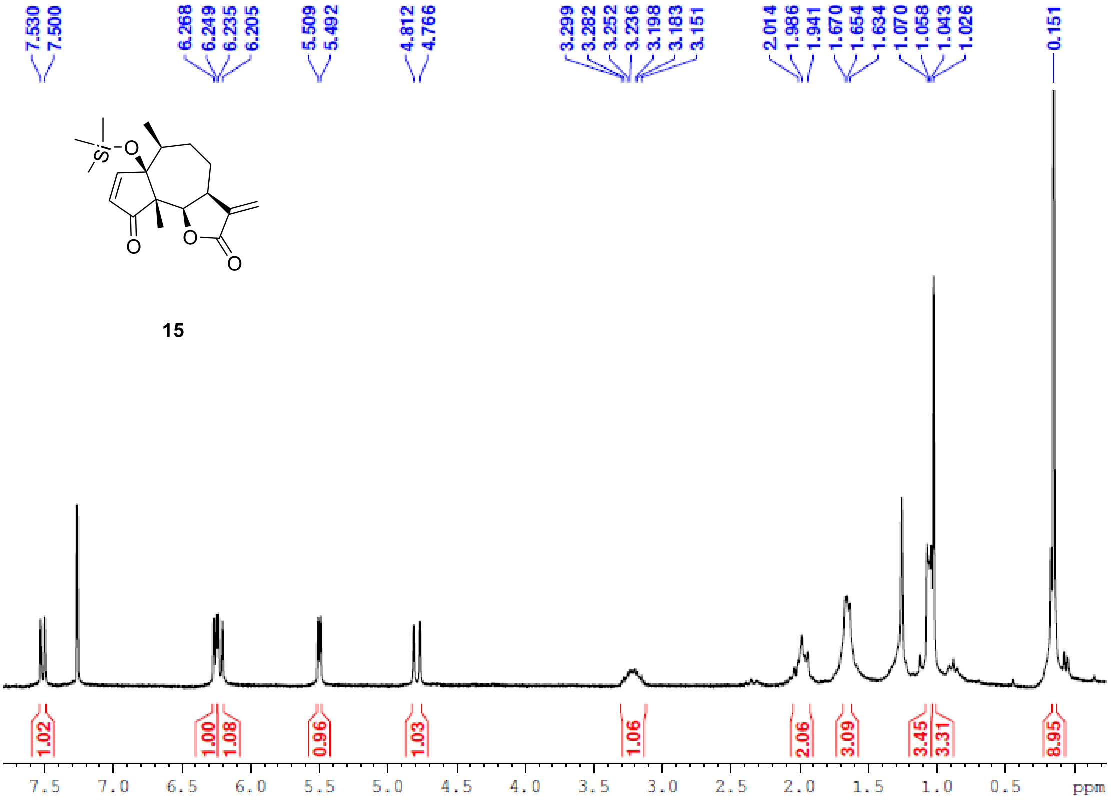

15

Figure S35. ^{1}H-NMR of **15**

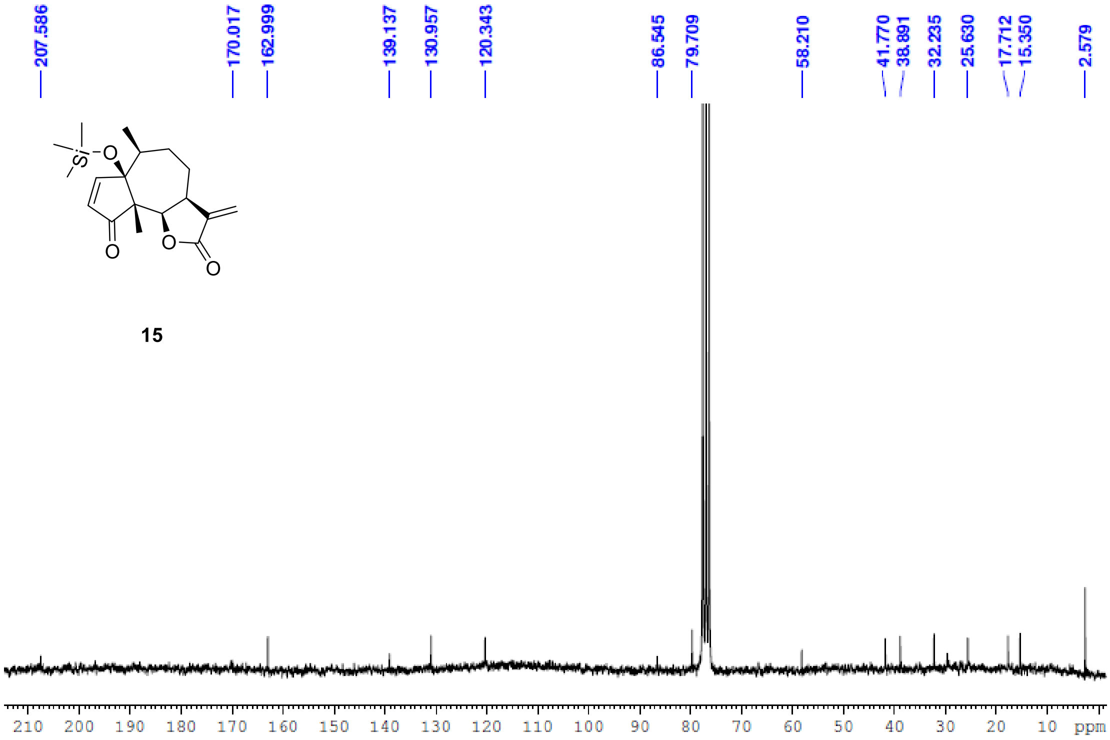

15

Figure S36. ^{13}C-NMR of **15**

5. HRMS-ES of compounds 1-15

Elemental Composition Report **Page 1**

Tolerance = 5.0 PPM / DBE: min = -10.0, max = 1000.0
Element prediction: Off
Number of isotope peaks used for i-FIT = 2

Monoisotopic Mass, Even Electron Ions
378 formula(e) evaluated with 1 results within limits (all results (up to 1000) for each mass)
Elements Used:
C: 0-60 H: 0-70 N: 0-1 O: 0-6 Na: 0-1 S: 0-2
(ESI 17-954) Osvaldo D (OJD-Cuma) 5 (0.225)

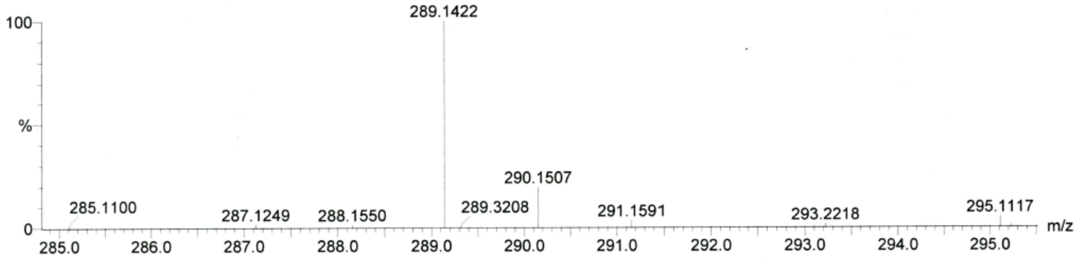

Minimum:	30.00				-10.0		
Maximum:	100.00		100.0	5.0	1000.0		
Mass	RA	Calc. Mass	mDa	PPM	DBE	i-FIT	Formula
289.1422	100.00	289.1416	0.6	2.1	4.5	8.4	C15 H22 O4 Na

Figure S37. HRMS-ES of **1**

Elemental Composition Report **Page 1**

Tolerance = 5.0 PPM / DBE: min = -10.0, max = 1000.0
Element prediction: Off
Number of isotope peaks used for i-FIT = 2

Monoisotopic Mass, Even Electron Ions
374 formula(e) evaluated with 1 results within limits (all results (up to 1000) for each mass)
Elements Used:
C: 0-60 H: 0-70 N: 0-1 O: 0-6 Na: 0-1 S: 0-2
(ESI 17-955) Osvaldo D (OJD-Helen) 102 (4.414)

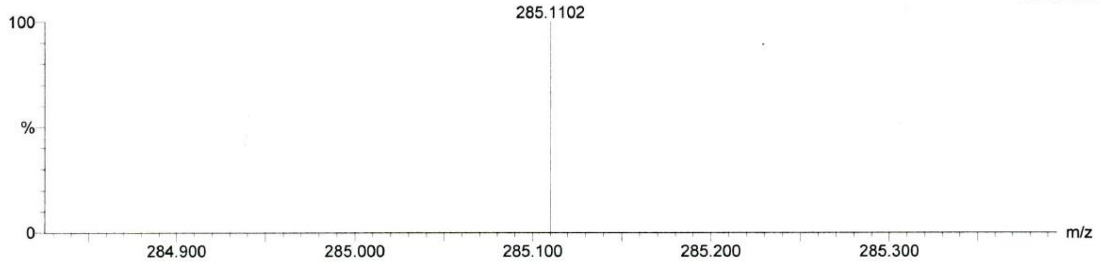

Minimum:				-10.0		
Maximum:		100.0	5.0	1000.0		
Mass	Calc. Mass	mDa	PPM	DBE	i-FIT	Formula
285.1102	285.1103	-0.1	-0.4	6.5	n/a	C15 H18 O4 Na

Figure S38. HRMS-ES of **2**

Elemental Composition Report **Page 1**

Tolerance = 5.0 PPM / DBE: min = -10.0, max = 1000.0
Element prediction: Off
Number of isotope peaks used for i-FIT = 2

Monoisotopic Mass, Even Electron Ions
378 formula(e) evaluated with 1 results within limits (all results (up to 1000) for each mass)
Elements Used:
C: 0-60 H: 0-70 N: 0-1 O: 0-6 Na: 0-1 S: 0-2
(ESI 17-954) Osvaldo D (OJD-Cuma) 5 (0.225) 1: TOF MS ES+ 5.96e+003

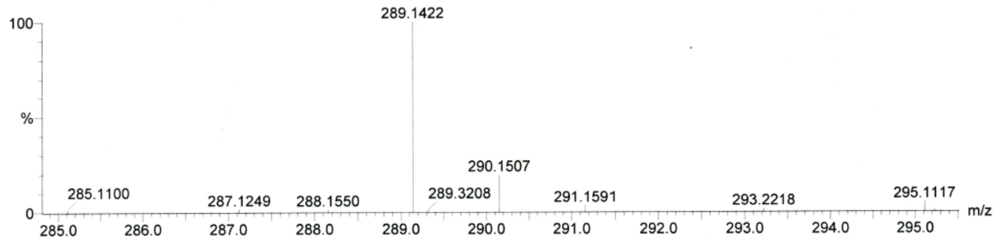

Minimum:	30.00				-10.0		
Maximum:	100.00		100.0	5.0	1000.0		
Mass	RA	Calc. Mass	mDa	PPM	DBE	i-FIT	Formula
289.1422	100.00	289.1416	0.6	2.1	4.5	8.4	C15 H22 O4 Na

Figure S39. HRMS-ES of **3**

Elemental Composition Report **Page 1**

Multiple Mass Analysis: 4 mass(es) processed
Tolerance = 5.0 PPM / DBE: min = -10.0, max = 1000.0
Element prediction: Off
Number of isotope peaks used for i-FIT = 2

Monoisotopic Mass, Even Electron Ions
4864 formula(e) evaluated with 19 results within limits (all results (up to 1000) for each mass)
Elements Used:
C: 0-53 H: 0-100 N: 0-1 O: 0-12 Na: 0-2 Si: 0-2
ESI (16-743) Maria F (MFB-008) 31 (1.340) 1: TOF MS ES+ 1.01e+004

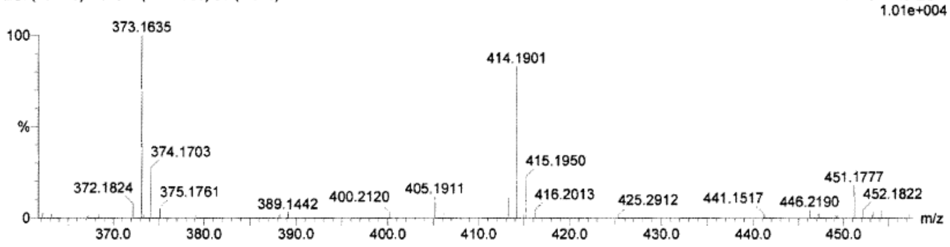

Minimum:	20.00				-10.0		
Maximum:	100.00		100.0	5.0	1000.0		
Mass	RA	Calc. Mass	mDa	PPM	DBE	i-FIT	Formula
373.1635	100.00	373.1634	0.1	0.3	-1.5	81.6	C13 H31 O7 Na2 Si
		373.1631	0.4	1.1	5.5	17.1	C18 H30 O3 Na Si2
		373.1627	0.8	2.1	6.5	50.2	C19 H26 O6 Na
		373.1624	1.1	2.9	13.5	33.6	C24 H25 O2 Si
		373.1651	-1.6	-4.3	9.5	18.1	C21 H25 O6
374.1703	27.33	374.1708	-0.5	-1.3	7.5	4.4	C20 H26 N O3 Na2
		374.1712	-0.9	-2.4	6.5	63.0	C19 H30 N Na2 Si2
		374.1694	0.9	2.4	-4.5	9.4	C9 H32 N O12 Si
414.1901	82.97	414.1900	0.1	0.2	-0.5	22.9	C15 H34 N O7 Na2 Si
		414.1897	0.4	1.0	6.5	43.7	C20 H33 N O3 Na Si2
		414.1893	0.8	1.9	7.5	9.4	C21 H29 N O6 Na
		414.1889	1.2	2.9	14.5	65.3	C26 H28 N O2 Si
		414.1917	-1.6	-3.9	10.5	0.7	C23 H28 N O6
		414.1921	-2.0	-4.8	9.5	76.8	C22 H32 N O3 Si2
415.1950	22.46	415.1951	-0.1	-0.2	-5.5	0.5	C12 H37 O10 Na2 Si
		415.1948	0.2	0.5	1.5	54.3	C17 H36 O6 Na Si2
		415.1944	0.6	1.4	2.5	3.1	C18 H32 O9 Na
		415.1941	0.9	2.2	9.5	67.2	C23 H31 O5 Si
		415.1968	-1.8	-4.3	5.5	10.5	C20 H31 O9

Figure S40. HRMS-ES of **4**

Elemental Composition Report

Multiple Mass Analysis: 3 mass(es) processed

Tolerance = 5.0 PPM / DBE: min = -10.0, max = 1000.0
Element prediction: Off
Number of isotope peaks used for i-FIT = 2

Monoisotopic Mass, Even Electron Ions
4085 formula(e) evaluated with 17 results within limits (all results (up to 1000) for each mass)
Elements Used:
C: 0-53 H: 0-100 N: 0-1 O: 0-12 Na: 0-2 Si: 0-2

ESI (16-744) Maria F (MFB-010 A) 19 (0.661)

2: TOF MS ES+
1.32e+004

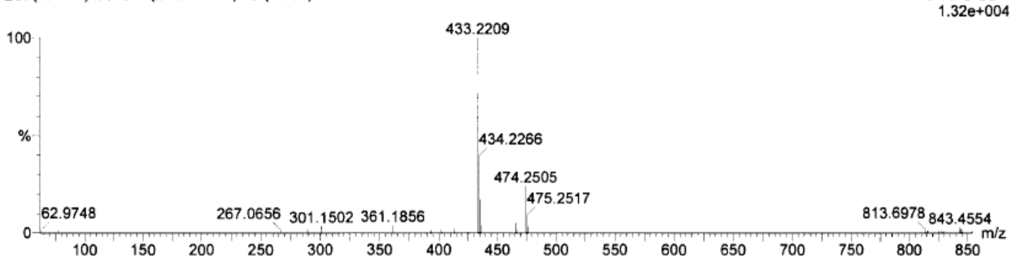

Minimum:	20.00				-10.0		
Maximum:	100.00		100.0	5.0	1000.0		

Mass	RA	Calc. Mass	mDa	PPM	DBE	i-FIT	Formula
433.2209	100.00	433.2210	-0.1	-0.2	-2.5	353.9	C16 H39 O8 Na2 Si
		433.2206	0.3	0.7	4.5	34.1	C21 H38 O4 Na Si2
		433.2202	0.7	1.6	5.5	284.3	C22 H34 O7 Na
		433.2199	1.0	2.3	12.5	17.5	C27 H33 O3 Si
		433.2226	-1.7	-3.9	8.5	197.0	C24 H33 O7
		433.2230	-2.1	-4.8	7.5	11.2	C23 H37 O4 Si2
434.2266	39.26	434.2256	1.0	2.3	10.5	39.4	C26 H34 N Na2 Si
		434.2280	-1.4	-3.2	13.5	22.6	C28 H33 N Na Si
		434.2283	-1.7	-3.9	6.5	155.4	C23 H34 N O4 Na2
		434.2287	-2.1	-4.8	5.5	33.8	C22 H38 N O Na2 Si2
474.2505	23.95	474.2507	-0.2	-0.4	-6.5	36.3	C14 H46 N O9 Na2 Si2
		474.2502	0.3	0.6	-5.5	142.0	C15 H42 N O12 Na2
		474.2499	0.6	1.3	1.5	25.9	C20 H41 N O8 Na Si
		474.2496	0.9	1.9	8.5	1.1	C25 H40 N O4 Si2
		474.2492	1.3	2.7	9.5	17.4	C26 H36 N O7
		474.2523	-1.8	-3.8	4.5	14.2	C22 H40 N O8 Si
		474.2526	-2.1	-4.4	-2.5	109.2	C17 H41 N O12 Na

Figure S41. HRMS-ES of **5**

Elemental Composition Report

Page 1

Multiple Mass Analysis: 2 mass(es) processed

Tolerance = 5.0 PPM / DBE: min = -10.0, max = 1000.0
Element prediction: Off
Number of isotope peaks used for i-FIT = 2

Monoisotopic Mass, Even Electron Ions
1492 formula(e) evaluated with 7 results within limits (all results (up to 1000) for each mass)
Elements Used:
C: 0-53 H: 0-100 O: 0-12 Na: 0-2 Si: 0-2

ESI (16-742) Maria F (MFB-011) 80 (2.791)

2: TOF MS ES+
4.05e+001

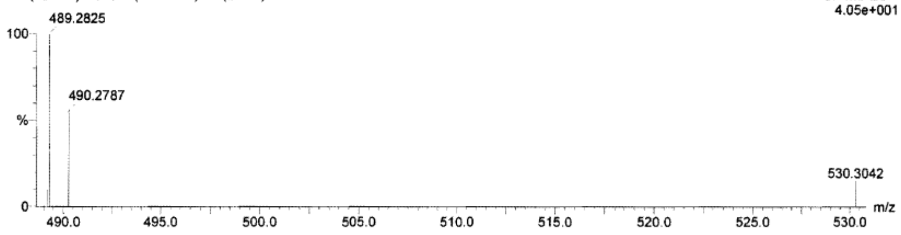

Minimum:	20.00				-10.0		
Maximum:	100.00		100.0	5.0	1000.0		

Mass	RA	Calc. Mass	mDa	PPM	DBE	i-FIT	Formula
489.2825	100.00	489.2825	0.0	0.0	12.5	0.7	C31 H41 O3 Si
		489.2828	-0.3	-0.6	5.5	2.1	C26 H42 O7 Na
		489.2832	-0.7	-1.4	4.5	0.8	C25 H46 O4 Na Si2
		489.2836	-1.1	-2.2	-2.5	2.4	C20 H47 O8 Na2 Si
		489.2808	1.7	3.5	1.5	1.1	C23 H47 O4 Na2 Si2
		489.2804	2.1	4.3	2.5	2.5	C24 H43 O7 Na2
		489.2801	2.4	4.9	9.5	0.9	C29 H42 O3 Na Si
490.2787	55.57	---					

Figure S42. HRMS-ES of **6**

Elemental Composition Report **Page 1**

Multiple Mass Analysis: 4 mass(es) processed

Tolerance = 5.0 PPM / DBE: min = -10.0, max = 1000.0
Element prediction: Off
Number of isotope peaks used for i-FIT = 2

Monoisotopic Mass, Even Electron Ions
6765 formula(e) evaluated with 29 results within limits (all results (up to 1000) for each mass)
Elements Used:
C: 0-53 H: 0-100 N: 0-1 O: 0-12 Na: 0-2 Si: 0-2
ESI (16-745) Maria F (MFB-012) 86 (2.997) 2: TOF MS ES+ 1.28e+004

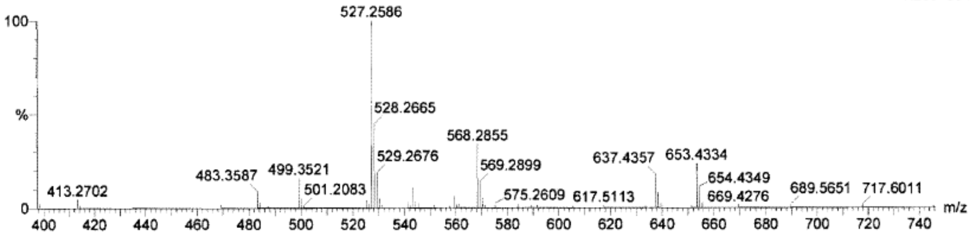

Minimum:	20.00				-10.0		
Maximum:	100.00		100.0	5.0	1000.0		

Mass	RA	Calc. Mass	mDa	PPM	DBE	i-FIT	Formula
527.2586	100.00	527.2586	0.0	0.0	20.5	9.3	C37 H35 O3
		527.2590	-0.4	-0.8	19.5	35.9	C36 H39 Si2
		527.2594	-0.8	-1.5	12.5	20.5	C31 H40 O4 Na Si
		527.2597	-1.1	-2.1	5.5	262.0	C26 H41 O8 Na2
		527.2601	-1.5	-2.8	4.5	37.2	C25 H45 O5 Na2 Si2
		527.2570	1.6	3.0	9.5	48.8	C29 H41 O4 Na2 Si
		527.2566	2.0	3.8	16.5	18.2	C34 H40 Na Si2
		527.2562	2.4	4.6	17.5	30.1	C35 H36 O3 Na
528.2665	44.59	528.2667	-0.2	-0.4	21.5	0.7	C38 H35 N Na
		528.2660	0.5	0.9	1.5	26.9	C21 H46 N O10 Si2
		528.2675	-1.0	-1.9	13.5	0.3	C32 H40 N O Na2 Si
		528.2643	2.2	4.2	18.5	0.9	C36 H36 N Na2
		528.2691	-2.6	-4.9	24.5	6.0	C40 H34 N
568.2855	34.03	568.2856	-0.1	-0.2	20.5	49.6	C38 H42 N Si2
		568.2852	0.3	0.5	21.5	4.7	C39 H38 N O3
		568.2859	-0.4	-0.7	13.5	1.6	C33 H43 N O4 Na Si
		568.2862	-0.7	-1.2	6.5	29.7	C28 H44 N O8 Na2
		568.2866	-1.1	-1.9	5.5	0.2	C27 H48 N O5 Na2 Si2
		568.2835	2.0	3.5	10.5	0.3	C31 H44 N O4 Na2 [illegible]

Figure S43. HRMS-ES of **7**

Elemental Composition Report **Page 1**

Tolerance = 5.0 PPM / DBE: min = -10.0, max = 1000.0
Element prediction: Off
Number of isotope peaks used for i-FIT = 2

Monoisotopic Mass, Even Electron Ions
416 formula(e) evaluated with 1 results within limits (all results (up to 1000) for each mass)
Elements Used:
C: 0-60 H: 0-70 N: 0-1 O: 0-6 Na: 0-1 S: 0-2
(ESI 17-957) Osvaldo D (OJD-F002) 10 (0.433) 1: TOF MS ES+ 1.94e+003

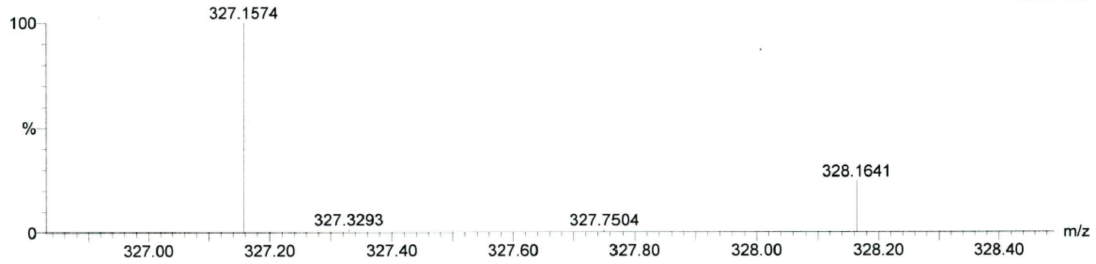

Minimum:	30.00				-10.0		
Maximum:	100.00		100.0	5.0	1000.0		

Mass	RA	Calc. Mass	mDa	PPM	DBE	i-FIT	Formula
327.1574	100.00	327.1572	0.2	0.6	6.5	5.1	C18 H24 O4 Na

Figure S44. HRMS-ES of **8**

Elemental Composition Report

Page 1

Tolerance = 5.0 PPM / DBE: min = -10.0, max = 1000.0
Element prediction: Off
Number of isotope peaks used for i-FIT = 2

Monoisotopic Mass, Even Electron Ions
450 formula(e) evaluated with 1 results within limits (all results (up to 1000) for each mass)
Elements Used:
C: 0-60 H: 0-70 N: 0-1 O: 0-6 Na: 0-1 S: 0-2
(ESI 17-956) Osvaldo D (OJD-F001) 21 (0.916)

1: TOF MS ES+
8.10e+003

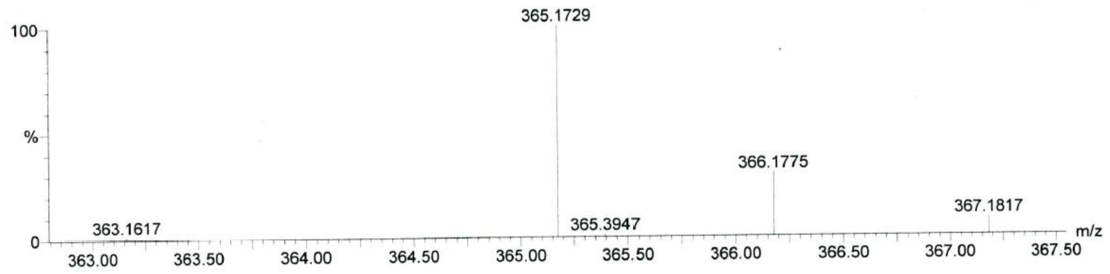

Minimum:	30.00				-10.0		
Maximum:	100.00		100.0	5.0	1000.0		
Mass	RA	Calc. Mass	mDa	PPM	DBE	i-FIT	Formula
365.1729	100.00	365.1729	0.0	0.0	8.5	41.3	C21 H26 O4 Na

Figure S45. HRMS-ES of **9**

Elemental Composition Report

Page 1

Tolerance = 5.0 PPM / DBE: min = -10.0, max = 1000.0
Element prediction: Off
Number of isotope peaks used for i-FIT = 2

Monoisotopic Mass, Even Electron Ions
1568 formula(e) evaluated with 6 results within limits (all results (up to 1000) for each mass)
Elements Used:
C: 0-120 H: 0-150 N: 0-6 O: 0-20 Na: 0-1
(ESI 17-986) Osvaldo (OJD-F 012-B) pos 78 (2.719)

2: TOF MS ES+
5.33e+002

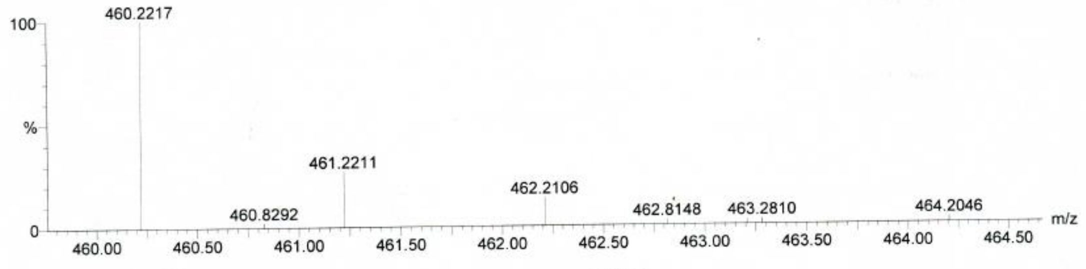

Minimum:	30.00				-10.0		
Maximum:	100.00		100.0	5.0	1000.0		
Mass	RA	Calc. Mass	mDa	PPM	DBE	i-FIT	Formula
460.2217	100.00	460.2217	0.0	0.0	-6.5	12.8	C12 H39 N O15 Na
		460.2212	0.5	1.1	11.5	0.7	C25 H31 N3 O4 Na
		460.2231	-1.4	-3.0	-1.5	8.0	C13 H35 N5 O11 Na
		460.2201	1.6	3.5	-7.5	19.4	C9 H38 N3 O17
		460.2236	-1.9	-4.1	14.5	2.0	C27 H30 N3 O4
		460.2196	2.1	4.6	10.5	0.0	C22 H30 N5 O6

Figure S46. HRMS-ES of **10**

Elemental Composition Report **Page 1**

Multiple Mass Analysis: 2 mass(es) processed
Tolerance = 5.0 PPM / DBE: min = -10.0, max = 1000.0
Element prediction: Off
Number of isotope peaks used for i-FIT = 2

Monoisotopic Mass, Even Electron Ions
2688 formula(e) evaluated with **14** results within limits (all results (up to 1000) for each mass)
Elements Used:
C: 0-100 H: 0-70 N: 0-6 O: 0-15 Na: 0-1
(ESI 17-977) Osvaldo D (OJD F 011-Y) 35 (1.223) 2: TOF MS ES+ 6.01e+002

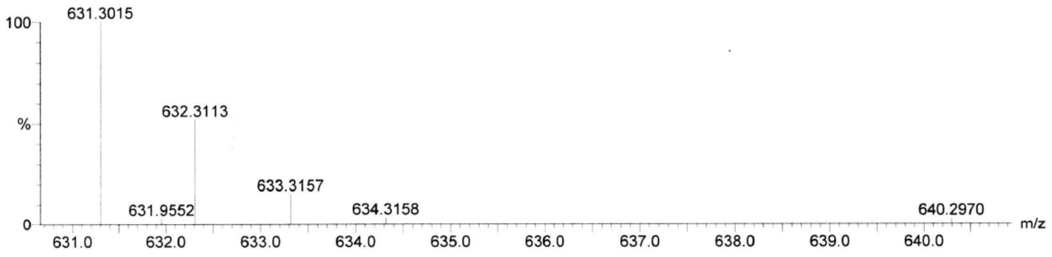

Minimum:	30.00				-10.0		
Maximum:	100.00		100.0	5.0	1000.0		

Mass	RA	Calc. Mass	mDa	PPM	DBE	i-FIT	Formula
631.3015	100.00	631.3014	0.1	0.2	0.5	30.3	C22 H48 N4 O15 Na
		631.3019	-0.4	-0.6	16.5	4.3	C36 H43 N2 O8
		631.3009	0.6	1.0	18.5	4.2	C35 H40 N6 O4 Na
		631.3001	1.4	2.2	29.5	0.4	C48 H39 O
		631.3033	-1.8	-2.9	21.5	2.4	C37 H39 N6 O4
		631.2995	2.0	3.2	13.5	6.7	C34 H44 N2 O8 Na
		631.3036	-2.1	-3.3	17.5	2.3	C39 H44 O6 Na
		631.3038	-2.3	-3.6	3.5	24.5	C24 H47 N4 O15
632.3113	51.59	632.3119	-0.6	-0.9	4.5	0.1	C25 H47 N5 O12 Na
		632.3124	-1.1	-1.7	20.5	8.4	C39 H42 N3 O5
		632.3100	1.3	2.1	17.5	6.6	C37 H43 N3 O5 Na
		632.3141	-2.8	-4.4	21.5	10.8	C42 H43 N O3 Na
		632.3084	2.9	4.6	16.5	4.7	C34 H42 N5 O7
		632.3143	-3.0	-4.7	7.5	0.7	C27 H46 N5 O12

Figure S47. HRMS-ES of **11**

Elemental Composition Report **Page 1**

Multiple Mass Analysis: 2 mass(es) processed
Tolerance = 5.0 PPM / DBE: min = -10.0, max = 1000.0
Element prediction: Off
Number of isotope peaks used for i-FIT = 2

Monoisotopic Mass, Even Electron Ions
2064 formula(e) evaluated with 6 results within limits (all results (up to 1000) for each mass)
Elements Used:
C: 0-53 H: 0-100 N: 0-1 O: 0-12 Na: 0-2 Si: 0-2
ESI (16-746) Maria F (MFB-020) 20 (0.696) 2: TOF MS ES+ 3.36e+003

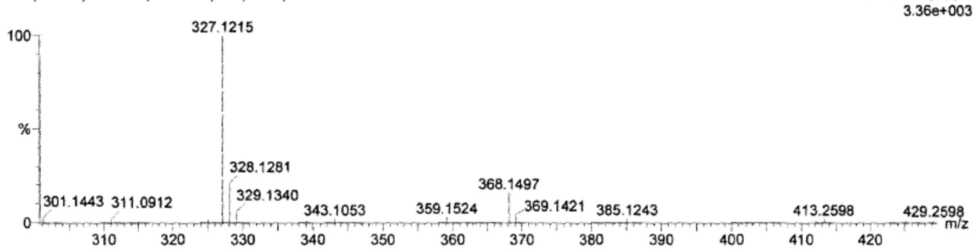

Minimum:	20.00				-10.0		
Maximum:	100.00		100.0	5.0	1000.0		

Mass	RA	Calc. Mass	mDa	PPM	DBE	i-FIT	Formula
327.1215	100.00	327.1216	-0.1	-0.3	-0.5	6.3	C11 H25 O6 Na2 Si
		327.1213	0.2	0.6	6.5	33.3	C16 H24 O2 Na Si2
		327.1208	0.7	2.1	7.5	1.7	C17 H20 O5 Na
		327.1205	1.0	3.1	14.5	46.5	C22 H19 O Si
328.1281	20.83	328.1275	0.6	1.8	-3.5	20.6	C7 H26 N O11 Si
		328.1289	-0.8	-2.4	8.5	4.4	C18 H20 N O2 Na2

Figure S48. HRMS-ES of **12**

Elemental Composition Report **Page 1**

Multiple Mass Analysis: 4 mass(es) processed
Tolerance = 5.0 PPM / DBE: min = -10.0, max = 1000.0
Element prediction: Off
Number of isotope peaks used for i-FIT = 2

Monoisotopic Mass, Even Electron Ions
4660 formula(e) evaluated with 19 results within limits (all results (up to 1000) for each mass)
Elements Used:
C: 0-53 H: 0-100 N: 0-1 O: 0-12 Na: 0-2 Si: 0-2
ESI (16-748) Maria F (MFB-022A) 13 (0.453) 2: TOF MS ES+
3.03e+003

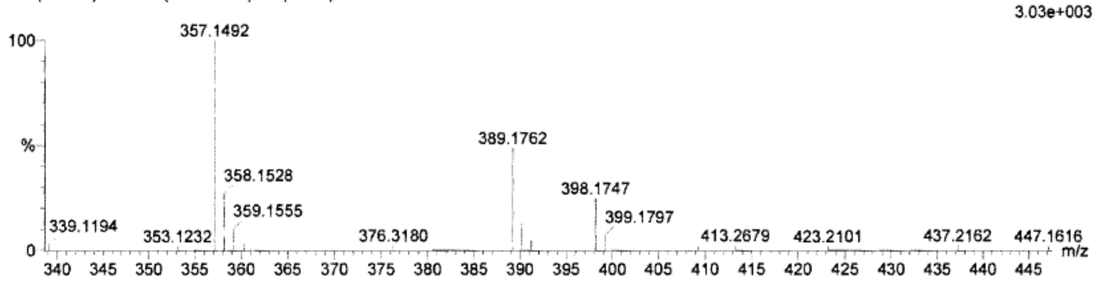

Minimum:	20.00				-10.0		
Maximum:	100.00		100.0	5.0	1000.0		
Mass	RA	Calc. Mass	mDa	PPM	DBE	i-FIT	Formula
357.1492	100.00	357.1491	0.1	0.3	14.5	0.1	C24 H21 O3
		357.1495	-0.3	-0.8	13.5	33.0	C23 H25 Si2
		357.1498	-0.6	-1.7	6.5	0.6	C18 H26 O4 Na Si
		357.1501	-0.9	-2.5	-0.5	70.0	C13 H27 O8 Na2
		357.1505	-1.3	-3.6	-1.5	3.4	C12 H31 O5 Na2 Si2
358.1528	26.77	358.1533	-0.5	-1.4	0.5	30.6	C12 H28 N O9 Si
		358.1541	-1.3	-3.6	-7.5	36.6	C6 H33 N O10 Na Si2
389.1762	48.69	389.1763	-0.1	-0.3	-1.5	22.9	C14 H31 O9 Na2
		389.1760	0.2	0.5	5.5	0.3	C19 H30 O5 Na Si
		389.1757	0.5	1.3	12.5	24.7	C24 H29 O Si2
		389.1768	-0.6	-1.5	-2.5	0.1	C13 H35 O6 Na2 Si2
		389.1753	0.9	2.3	13.5	1.5	C25 H25 O4

Figure S49. HRMS-ES of **13**

Elemental Composition Report **Page 1**

Multiple Mass Analysis: 2 mass(es) processed
Tolerance = 5.0 PPM / DBE: min = -10.0, max = 1000.0
Element prediction: Off
Number of isotope peaks used for i-FIT = 2

Monoisotopic Mass, Even Electron Ions
2386 formula(e) evaluated with 10 results within limits (all results (up to 1000) for each mass)
Elements Used:
C: 0-53 H: 0-100 N: 0-1 O: 0-12 Na: 0-2 Si: 0-2
ESI (16-749) Maria F (MFB-025) 28 (0.976) 2: TOF MS ES+
3.28e+002

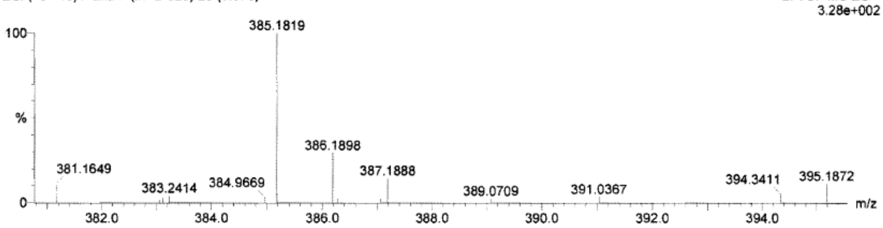

Minimum:	20.00				-10.0		
Maximum:	100.00		100.0	5.0	1000.0		
Mass	RA	Calc. Mass	mDa	PPM	DBE	i-FIT	Formula
385.1819	100.00	385.1818	0.1	0.3	-1.5	0.5	C14 H35 O5 Na2 Si2
		385.1814	0.5	1.3	-0.5	7.3	C15 H31 O8 Na2
		385.1811	0.8	2.1	6.5	0.2	C20 H30 O4 Na Si
		385.1808	1.1	2.9	13.5	3.1	C25 H29 Si2
		385.1804	1.5	3.9	14.5	0.1	C26 H25 O3
		385.1835	-1.6	-4.2	9.5	0.1	C22 H29 O4 Si
386.1898	29.40	386.1892	0.6	1.6	7.5	3.3	C21 H30 N O Na2 Si
		386.1909	-1.1	-2.8	18.5	2.1	C29 H24 N
		386.1885	1.3	3.4	15.5	2.8	C27 H25 N Na
		386.1916	-1.8	-4.7	10.5	2.6	C23 H29 N O Na Si

Figure S50. HRMS-ES of **14**

Elemental Composition Report

Multiple Mass Analysis: 2 mass(es) processed
Tolerance = 5.0 PPM / DBE: min = -10.0, max = 1000.0
Element prediction: Off
Number of isotope peaks used for i-FIT = 2

Monoisotopic Mass, Even Electron Ions
2230 formula(e) evaluated with 9 results within limits (all results (up to 1000) for each mass)
Elements Used:
C: 0-53 H: 0-100 N: 0-1 O: 0-12 Na: 0-2 Si: 0-2
ESI (16-750) Maria F (MFB-040) 25 (0.870) 2: TOF MS ES+ 1.27e+003

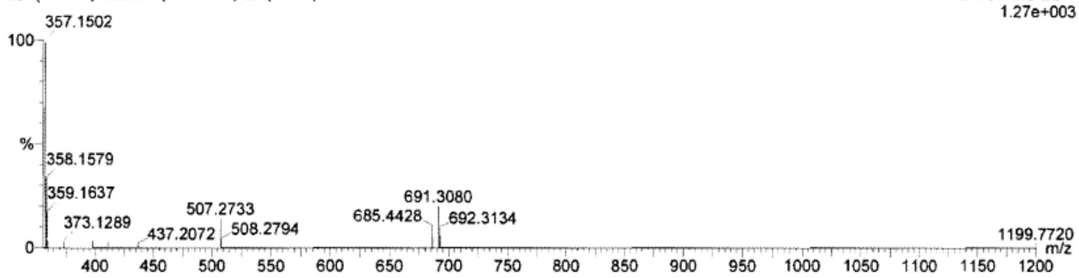

Minimum:	20.00				-10.0		
Maximum:	100.00		100.0	5.0	1000.0		
Mass	RA	Calc. Mass	mDa	PPM	DBE	i-FIT	Formula
357.1502	100.00	357.1501	0.1	0.3	-0.5	60.9	C13 H27 O8 Na2
		357.1505	-0.3	-0.8	-1.5	15.0	C12 H31 O5 Na2 Si2
		357.1498	0.4	1.1	6.5	10.6	C18 H26 O4 Na Si
		357.1495	0.7	2.0	13.5	1.1	C23 H25 Si2
		357.1491	1.1	3.1	14.5	7.0	C24 H21 O3
358.1579	33.71	358.1579	0.0	0.0	7.5	22.5	C19 H26 N O Na2 Si
		358.1572	0.7	2.0	15.5	19.6	C25 H21 N Na
		358.1565	1.4	3.9	-4.5	38.5	C8 H32 N O10 Si2
		358.1596	-1.7	-4.7	18.5	15.7	C27 H20 N

Figure S51. HRMS-ES of **15**
